# Age and sex effects across the blood proteome after ionizing radiation exposure can bias biomarker screening and risk assessment

**DOI:** 10.1038/s41598-022-10271-3

**Published:** 2022-04-29

**Authors:** Britta Langen, Egor Vorontsov, Johan Spetz, John Swanpalmer, Carina Sihlbom, Khalil Helou, Eva Forssell-Aronsson

**Affiliations:** 1 Department of Radiation Physics, Institute of Clinical Sciences, Sahlgrenska Cancer Center, Sahlgrenska Academy, University of Gothenburg, Gothenburg, Sweden; 2 Proteomics Core Facility, Sahlgrenska Academy, University of Gothenburg, Gothenburg, Sweden; 3 Department of Medical Physics and Biomedical Engineering, Sahlgrenska University Hospital, Gothenburg, Sweden; 4 Department of Oncology, Institute of Clinical Sciences, Sahlgrenska Cancer Center, Sahlgrenska Academy, University of Gothenburg, Gothenburg, Sweden; 5 Section of Molecular Radiation Biology, Department of Radiation Oncology, University of Texas Southwestern Medical Center, Dallas, TX 75390 USA

**Keywords:** Biomarkers, Preclinical research, Radiotherapy

## Abstract

Molecular biomarkers of ionizing radiation (IR) exposure are a promising new tool in various disciplines: they can give necessary information for adaptive treatment planning in cancer radiotherapy, enable risk projection for radiation-induced survivorship diseases, or facilitate triage and intervention in radiation hazard events. However, radiation biomarker discovery has not yet resolved the most basic features of personalized medicine: age and sex. To overcome this critical bias in biomarker identification, we quantitated age and sex effects and assessed their relevance in the radiation response across the blood proteome. We used high-throughput mass spectrometry on blood plasma collected 24 h after 0.5 Gy total body irradiation (15 MV nominal photon energy) from male and female C57BL/6 N mice at juvenile (7-weeks-old) or adult (18-weeks-old) age. We also assessed sex and strain effects using juvenile male and female BALB/c nude mice. We showed that age and sex created significant effects in the proteomic response regarding both extent and functional quality of IR-induced responses. Furthermore, we found that age and sex effects appeared non-linear and were often end-point specific. Overall, age contributed more to differences in the proteomic response than sex, most notably in *immune responses*, *oxidative stress*, and *apoptotic cell death*. Interestingly, sex effects were pronounced for *DNA damage and repair* pathways and associated cellular outcome (pro-survival vs. pro-apoptotic). Only one protein (AHSP) was identified as a potential general biomarker candidate across age and sex, while GMNN, REG3B, and SNCA indicated some response similarity across age. This low yield advocated that unisex or uniage biomarker screening approaches are not feasible. In conclusion, age- and sex-specific screening approaches should be implemented as standard protocol to ensure robustness and diagnostic power of biomarker candidates. Bias-free molecular biomarkers are a necessary progression towards personalized medicine and integral for advanced adaptive cancer radiotherapy and risk assessment.

## Introduction

Personalized medicine has been on the horizon for almost two decades, but only few disciplines have entered this new era^1,2^. In cancer radiotherapy, so-called precision radiation oncology is still far from the bedside^2^. Similarly, personalized risk assessment of ionizing radiation (IR) exposure is needed but still in the early stages of research and development: applications range from adaptive cancer radiotherapy and risk projection of survivorship diseases^2,3^, over triage and treatment in radiation hazard events^4,5^, to manned spaceflight^6–8^. The keystone of personalized medicine is improved diagnostics that are sensitive to biological diversity and heterogeneity in respective conditions. This can be implemented with molecular biomarkers that indicate absorbed dose or resulting effects in irradiated tissue^3^. Biomarkers such as proteins, nucleic acids, or metabolites can be used to determine absorbed dose, monitor the therapeutic response in malignancies, or assess risk of radiation-induced survivorship diseases. In the past decade, advances have been made in genome-wide biomarker screening of radiation effects: for instance, a panel of biomarker genes with high diagnostic power was identified in human blood in a comprehensive meta-study^5^. Using animal models, tissue-specific biomarker candidates have also been proposed for external irradiation with e.g. x-rays^9,10^, gamma rays^11,12^, or neutrons^10,13^, and for internal exposure from various radionuclides^14–25^.

High-throughput protein analysis emerged as a dedicated discipline in the mid-90 s^26,27^. Since, proteomics has been mainly used in radiation research for in vitro studies or malignant disease while blood-based biomarker screening remained underexplored; presumably the first study to apply this approach was published in 2009 and compared protein expression profiles in mouse blood plasma after several days following acute total body exposure to 3 Gy absorbed dose from ^137^Cs gamma rays^28^. It should be noted that relatively few proteins were differentially abundant between control and exposure groups (19 and 29 proteins at days 2 and 7, respectively), which may be owed to the initial 2D gel electrophoresis targeting high expression proteins. Further examples of blood-based screening in the total body exposure setting are plasma from non-human primates irradiated with ^60^Co gamma rays at absorbed doses of 1–8.5 Gy^29^, or 6.7 and 7.4 Gy^30^, or a humanized mouse model irradiated with 0–2 Gy from X-rays^31^. While these studies identified valuable protein markers for respective exposure conditions, their validity for the other sex or different age groups is undetermined. In the clinical setting, advances have been made to utilize blood plasma (or serum) proteomics for dosimetry or prediction of radiation responses^32–34^, but age and sex are not employed as distinctive features that can influence proteomic responses.

Ironically, on the path to personalized medicine, the basic features that stratify a cohort and influence outcome have remained largely unaddressed in biomarker screening: age and sex^35^. Both features are easily categorized and have comparatively few functional subgroups (i.e. several development stages and sex groups), which makes their translation to the bedside highly cost-effective and time-efficient. Even in research, age and sex have been neglected as basic experimental parameters across most biological disciplines^36^. The vast majority of articles has not been analyzed by sex–and if, they have a strong male bias. Although radiobiology was not included as a discipline, related cross-disciplinary fields such as immunology and pharmacology only had around 5% and 20% of articles with sex-specific cohorts, respectively. Considering age, the issue becomes even more complex: most studies use single-age cohorts where the age of the animal is chosen for practical reasons. Moreover, relating age between animal and human is difficult due to their different progression through development stages^37^.

It is well-established in principle that age and sex influence the radiation response, but the molecular basis is still poorly understood. This lack of knowledge contributes to low-resolution dose limits and uncertainties in current risk estimates^38,39^. Dose limits have not yet been established with sex-specific regimens by most authorities, although male and female reference phantoms have been developed for research and are recommended^39^. Similarly, differences in radiation sensitivity are described for prenatal development up to adolescence for various cancerous and non-cancerous malignancies^40,41^. However, dose recommendations rarely differentiate between the periods of human age past early adulthood^38^. Concerning radiation biomarker screening in particular, next-gen technology is highly sensitive to slight variations in dose–response relationships. The generalization of dose–response relationships across age- and sex-specific variations can severely compromise the sensitivity, specificity, and accuracy of biomarkers. The result would be reduced robustness and diagnostic power, which can lead to erroneous decision making in treatment planning or risk assessment and, ultimately, harm the patient. Biomarkers for juveniles are crucial in pediatric radiotherapy to monitor and counteract long-term complications or secondary cancer induction due to the long remaining natural lifespan. On the other hand, late adulthood (45–60) or early old age (60–75 years) are the predominant groups in cancer radiotherapy and should thus be accurately represented in pre-clinical research. However, the vast majority of biomarker screening studies have not addressed age and sex as critical parameters–neither as individual features nor in conjunction.

The underrepresentation of certain age or sex groups can produce several types of bias depending on the study design, such as exclusion bias, selection bias, or spectrum bias. Furthermore, intra-group variability can differ and thus confound the perceived variance of the response-signal from a biomarker candidate. In this exploratory screening study, we use the terms ‘sex effects’ and ‘age effects’ to denote the biological differences in responses which can cause any of the biases above if generalized or misrepresented.

To the best of our knowledge, age and sex effects have not been studied on the proteomic scale in context with blood-based biomarker screening. Here, we present proteomic data on the acute radiation response in blood plasma using a comprehensive cohort of juvenile and adult and male and female mice. In addition, we studied sex effects in conjunction with strain for a nude mouse model commonly used in tumor xenografting. Our aim was to determine the extent and quality of age and sex effects across the proteome for various analytical endpoints that are routinely used in omics-based screening studies. The overall purpose was to substantiate the knowledge base for biomarker screening, translational research, and risk assessment.

## Results

### Extent of age and sex effects differs between low and high abundance change proteins

**Figure 1:**
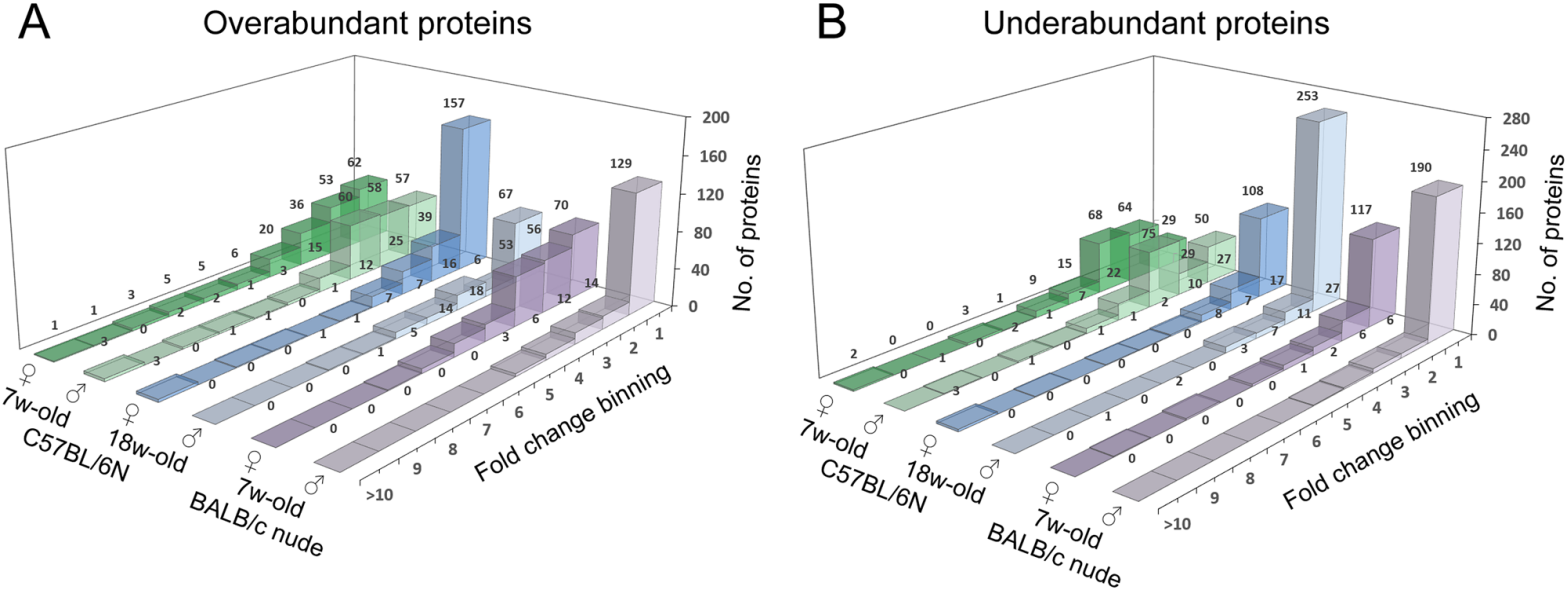
Fold change distribution of all filtered proteins. Histogram plots show the number (no.) of filtered proteins (i.e. accessions) with differential changes in overabundance (**A**) or underabundance (**B**) in each group in blood plasma 24 h after 0.5 Gy total body irradiation. The fold change was binned in onefold intervals from onefold up to tenfold or higher. 7w-old, 7-weeks-old (juvenile mice); 18w-old, 18-weeks-old (adult mice). ♀, female; ♂, male. Please note that presented data is normalized to respective unirradiated control groups.

**Figure 2:**
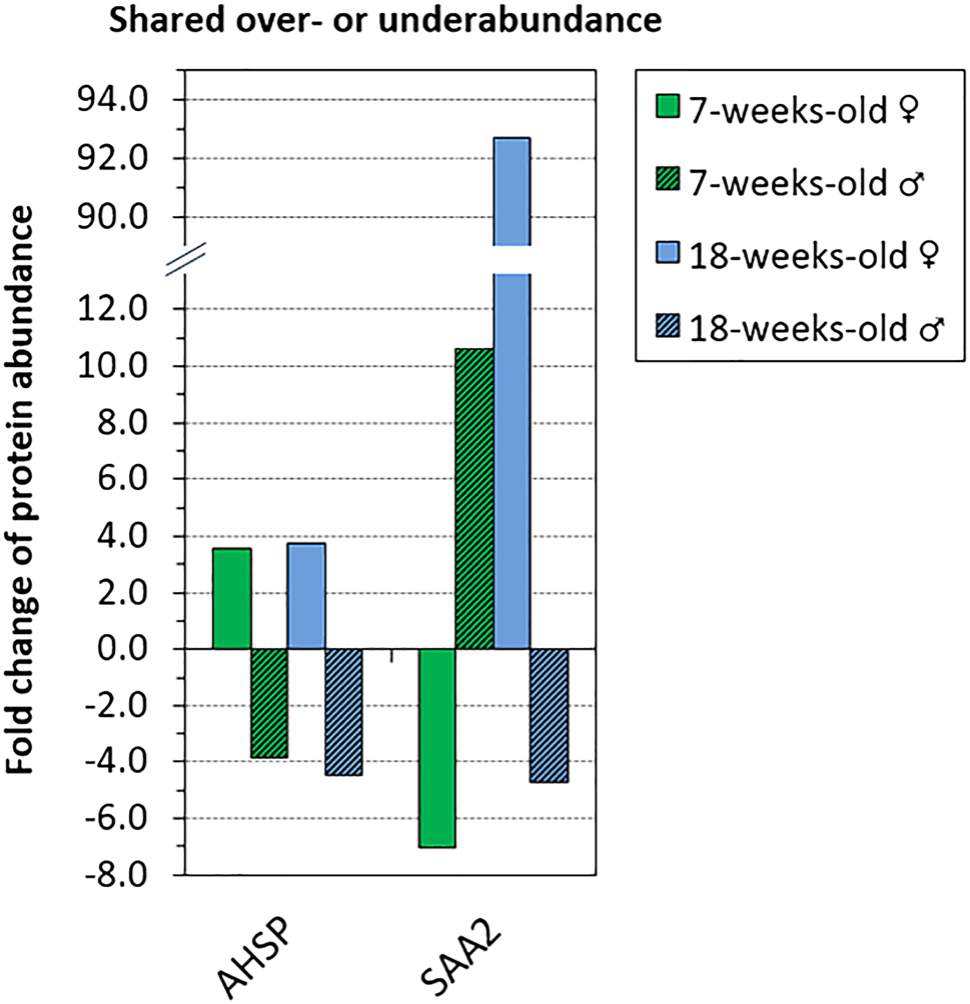
High-abundance change proteins shared between all age and sex groups. Twenty-four hours after 0.5 Gy total body irradiation, only two differentially abundant proteins with a fold change (FC) value |FC|≥ 3 were shared between female (♀, solid fill) and male (♂ hashed) C57BL/6 N mice at juvenile (7-weeks-old, green) and adult (18-weeks-old, blue) age in blood plasma. Alpha hemoglobin stabilizing protein (AHSP) showed similar FC values across age after irradiation, but inverted abundance across sex. Serum amyloid A-2 protein (SAA2) showed large FC variance across both age and sex. Please note that presented data is normalized to respective unirradiated control groups.

On the proteomic scale, 0.5 Gy total body irradiation induced a similar bulk response in mice of different age, sex, and strain. The number of differentially abundant proteins ranged between 369 and 392 for all groups (cf. Fig. 1). The distribution across low- and high-abundance change proteins, however, differed distinctly between age and strain for both overabundance (Fig. 1A) and underabundance (Fig. 1B). Sex differences, however, were often marginal in this regard. Excluding low-abundance change proteins (|FC|< 3), 175, 194, 61, 47, 115, and 30 proteins were differentially abundant (|FC|≥ 3) in juvenile black females and males, adult black females and males, and nude (juvenile) females and males, respectively (cf. Fig. 1). The greatest impact on high-abundance change proteins (|FC|≥ 3) was found to be age rather than sex in black mice, whereas sex-based difference remained pronounced in nude (juvenile) mice. Only two high-abundance change proteins (AHSP and SAA2) were detected in all age and sex groups (Fig. 2). AHSP showed similar response across age but abundance change was inverted across sex. In contrast, SAA2 showed no apparent age- or sex-dependent response, but a strong FC range across groups.

**Figure 3:**
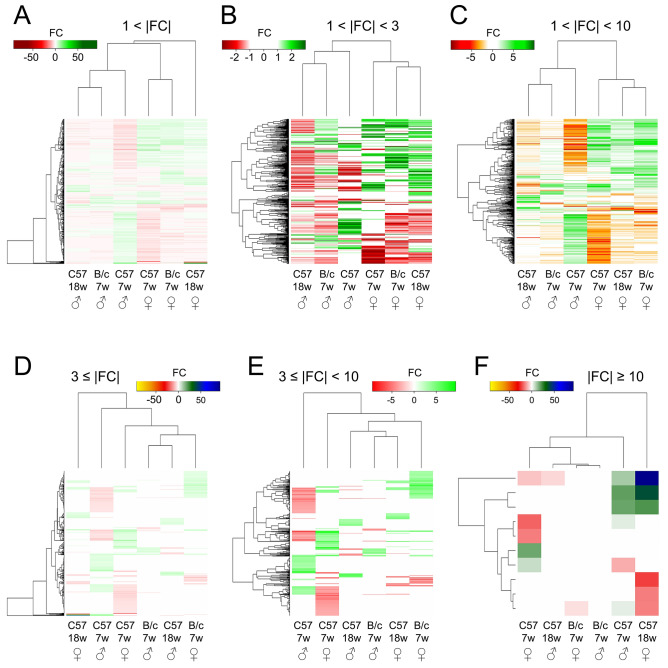
Relative distance between age, sex and strain groups in dependence of fold change intervals. Hierarchical cluster analysis of filtered proteins according to fold (FC) intervals of differentially abundant proteins in blood plasma 24 h after 0.5 Gy total body irradiation. Cutoff FC values were used to differentiate between the impact on distance of proteins with low (1 <|FC|< 3), high (3 ≤|FC|< 10), and very-high (|FC|≥ 10) abundance change: entire filtered cohort (**A**), low abundance change (**B**), low and high abundance change (**C**), high-to-very-high abundance change (**D**), high abundance change only (**E**), very-high abundance change only (**F**). Green, overabundance; red, underabundance; C57, C57BL/6 N mice; B/c, BALB/c nude mice; 7w, 7-weeks-old (juvenile) mice; 18w, 18-weeks-old (adult) mice; ♀, female; ♂, male. Please note that presented data is normalized to respective unirradiated control groups.

We used hierarchical clustering to analyze the extent of similarity of the bulk response between groups (Fig. 3). For all differentially abundant proteins (|FC|> 1), the sex dimension created a clear separation across age and strain (Fig. 3A). The clustering by sex was maintained for increasing FC range up to |FC|< 10 proteins (Fig. 3B,C). However, when low-abundance change proteins (|FC|< 3) were excluded, clear separation by age or sex disappeared (Fig. 3D); this was also the case when very-high abundance change proteins (|FC|≥ 10) were excluded (Fig. 3E). Regarding |FC|≥ 10 proteins only, males grouped with females within their strains but across different ages (Fig. 3F). In contrast with bulk differential abundance (cf. Fig. 1), sex had a larger impact than age (or strain) on hierarchical clustering.

### Age, not sex, is the larger contributor to functional effects in the proteomic radiation response

**Figure 4:**
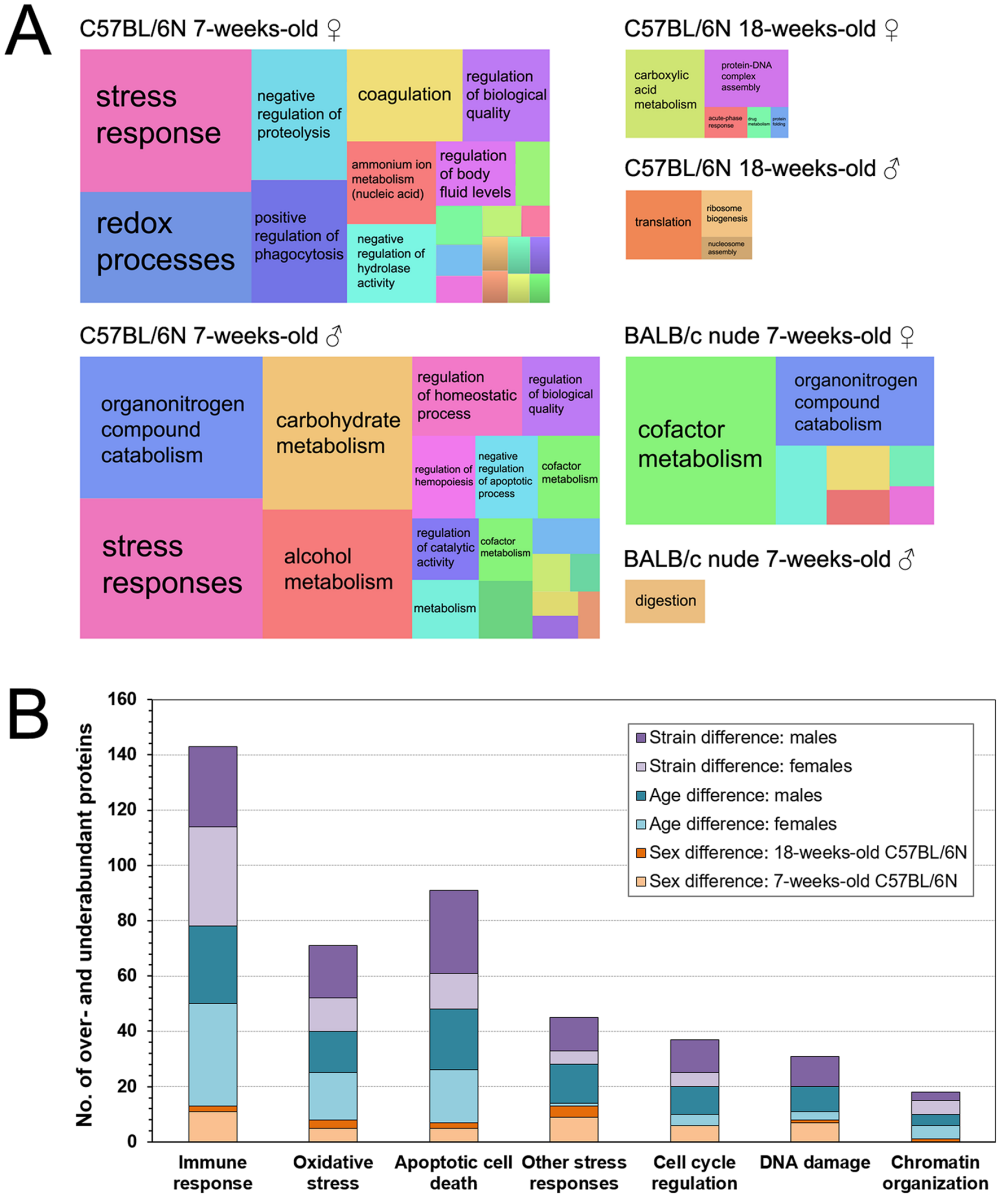
Biological quality of age, sex, and strain differences. Functional analysis of proteins with differential abundance (|FC|≥ 3) in blood plasma 24 h after 0.5 Gy total body irradiation. Treemap (**A**) shows functional association of the radiation-induced proteomic changes using REVIGO (see treemap header for group info). Note that treemap size is scaled according to the number of differentially abundant proteins in each group to allow for relative comparisons. The relative difference in over- and underabundant proteins (cumulative) is shown in paired comparisons (**B**), i.e. between males and females aged 7-weeks-old (juveniles, light orange) or 18-weeks-old (adults, orange), between 7-weeks-old and 18-weeks-old females (cyan) or males (dark cyan), and between C57BL/6 N and BALB/c nude strains (all 7-weeks-old) being either female (light lilac) or male (lilac). ♀, female; ♂, male. Please note that presented data is normalized to respective unirradiated control groups.

We used enrichment analyses (|FC|≥ 3) to assess the functional quality of the proteomic response after 0.5 Gy total body irradiation. The biological quality appeared heterogeneous across age, sex and strain (Fig. 4A). (Detailed functional associations are given in SI Appendix, Fig. S1). Functional similarities were only apparent between the sexes to some extent: juvenile black females and males shared *stress responses* and *apoptosis/phagocytosis*, but otherwise diverged. While adult black females and males had only few enriched processes, they did show some similarity in *DNA-nucleosome assembly*. In nude (juvenile) females and males, no apparent similarities were observed.

In order to discern IR-associated responses from general enrichment, we manually filtered proteins associated with GO terms for hallmarks of IR-induced processes. The number of differentially abundant proteins associated with respective processes are given in SI Appendix, Table S1. Briefly, a clear sex-specific trend was found for *immune response* and *oxidative stress*, where females showed more pronounced responses than males irrespective of age or strain. For the other processes, no consistent correlation with sex was observed. We plotted pair-wise comparisons (difference of number of associated proteins; cf. SI Appendix, Table S1) to quantify the relative impact of age, sex, and strain in mutual dependence (Fig. 4B). Overall, sex effects were low. For *immune response*, *oxidative stress*, and *apoptotic cell death*, sex effects were marginal for both juvenile and adult (black) mice, whereas age and strain had a strong influence on response intensity in either sex. For *other cellular stress*, *cell cycle regulation*, *DNA damage and repair*, and *chromatin organization*, age and strain had a generally stronger impact in males than in females, but the extent was less pronounced. Overall, sex had the lowest impact on functional enrichment analysis, which was at the lowest in adult mice. Interestingly, male mice were subject to the largest overall confounder by strain, followed by age.

### Age and sex strongly influence the regulatory quality of IR-associated processes

**Figure 5:**
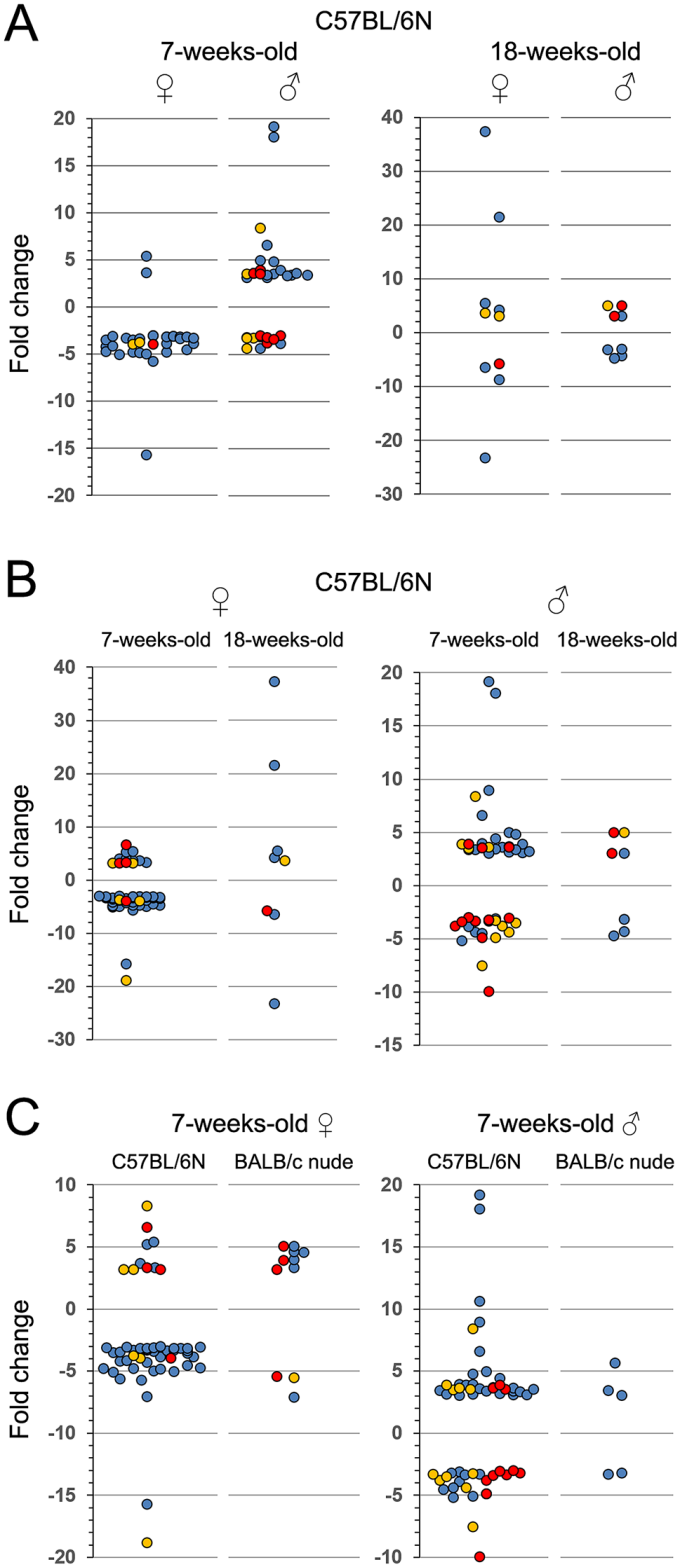
Pairwise comparison of fold change distribution for differentially abundant proteins unique for each age, sex and strain group. Proteins (dots) with differential abundance (|FC|≥ 3) in blood plasma 24 h after 0.5 Gy total body irradiation were associated with Gene Ontology terms; selected ionizing radiation-induced responses are shown, i.e. *immune response* (blue), *cell cycle* (yellow), and *DNA damage and repair* (red). Sex difference for each age group (**A**), age difference for each sex (**B**), and strain difference for each sex (juveniles, 7-weeks-old) (**C**). Note difference in scaling of y-axis. ♀, female; ♂, male. Please note that presented data is normalized to respective unirradiated control groups.

**Figure 6:**
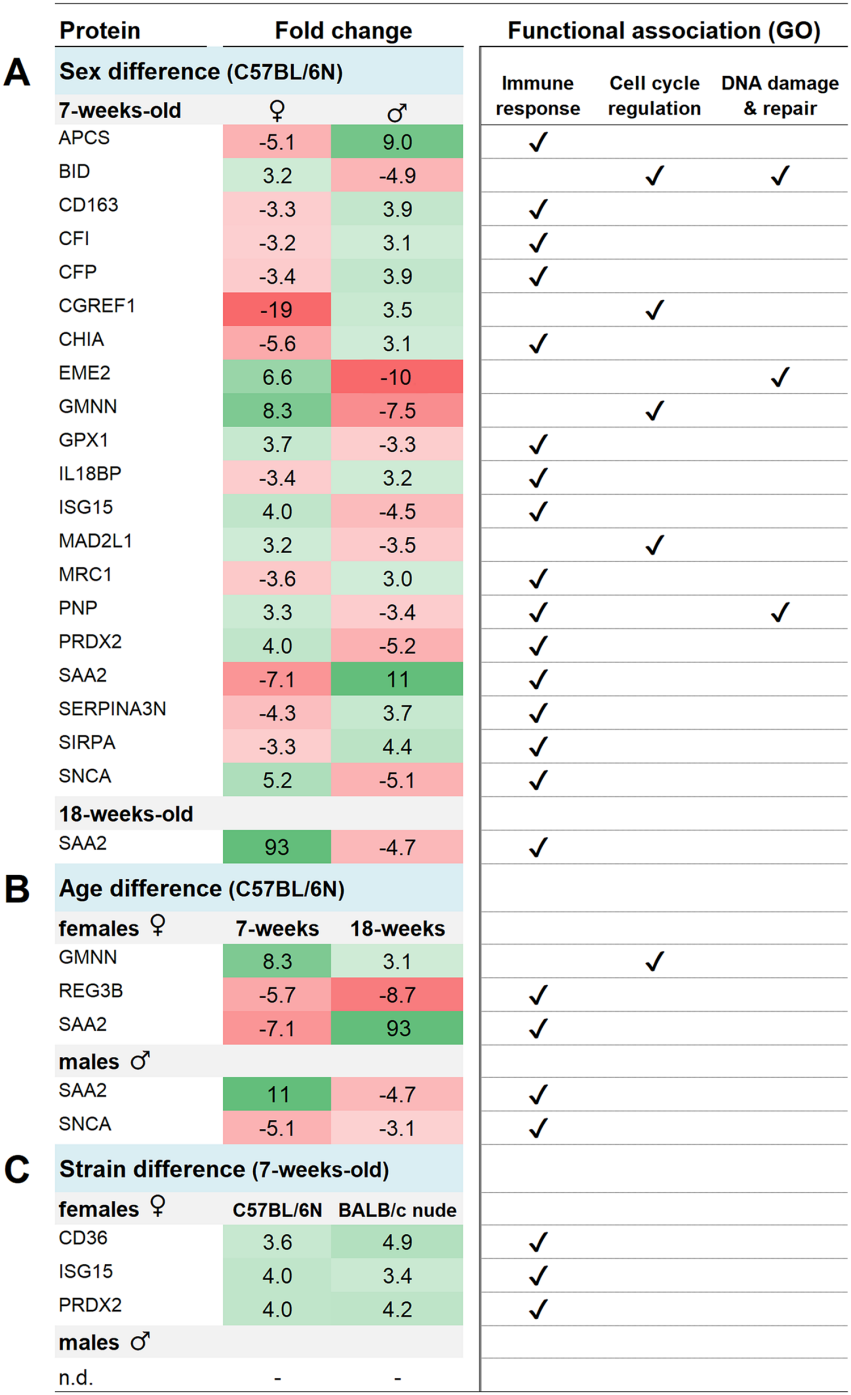
Panel of differentially abundant proteins shared between sex, age or strain in paired comparison. Proteins with differential abundance (|FC|≥ 3) in blood plasma 24 h after 0.5 Gy total body irradiation were associated with Gene Ontology terms (right-hand panel); selected radiation-induced responses are shown, i.e. *immune response, cell cycle*, and *DNA damage and repair*. Protein lists given as pairwise comparison between both sexes (**A**), both age groups (**B**), and both strains (**C**). ♀, female; ♂, male; n.d., none determined. Please note that presented data is normalized to respective unirradiated control groups.

We analyzed differential abundance profiles (|FC| cutoff ≥ 3) for *DNA damage and repair*, *cell cycle regulation*, and *immune responses* in detail as hallmarks of ionizing radiation responses*.* Overall, unique proteins not shared between groups for these processes (Fig. 5) were generally more frequent than shared proteins (Fig. 6). Moreover, unique proteins showed different distribution profiles (over- vs. underabundance) or different dynamic range (FC values) for the same processes when compared between groups. Sex effects were pronounced in black mice but the extent and dynamic range appeared process-specific and differed between juvenile and adult mice (Fig. 5A). Age-specific differences were apparent for both sexes and all investigated processes in black mice. Interestingly, the feature of age-dependent dynamic range appeared sex-specific: in females, adult mice showed a larger FC range despite the low overall differential protein abundance (Fig. 5B, left panel), whereas in males, juvenile mice showed both a larger FC range and number of differentially abundant proteins (Fig. 5B, right panel).

Strain-specific differences were distinct in both sexes for almost all processes (Fig. 5C). Interestingly, *DNA damage and repair* in females was the only process that responded in a similar manner across strains. Regarding shared proteins, differential abundance was generally inverted in almost all pair-wise comparisons (Fig. 6A–C). The only shared proteins with same over- or underabundance patterns (albeit different FC levels) in juvenile and adult black mice were GMNN and REG3B in females and SNCA in males (Fig. 6B). Black and nude females shared CD36, ISG15, and PRDX2 with similar FC values (Fig. 6C).

Taken together, radiation induced rather unique pathway responses for the different sex, age and strain groups not only resulting in a larger panel of unique differential proteins, but also in disparate (inverted) differential abundance if the same proteins were detected.

### Males respond stronger in DNA damage and repair irrespective of age or strain

**Figure 7:**
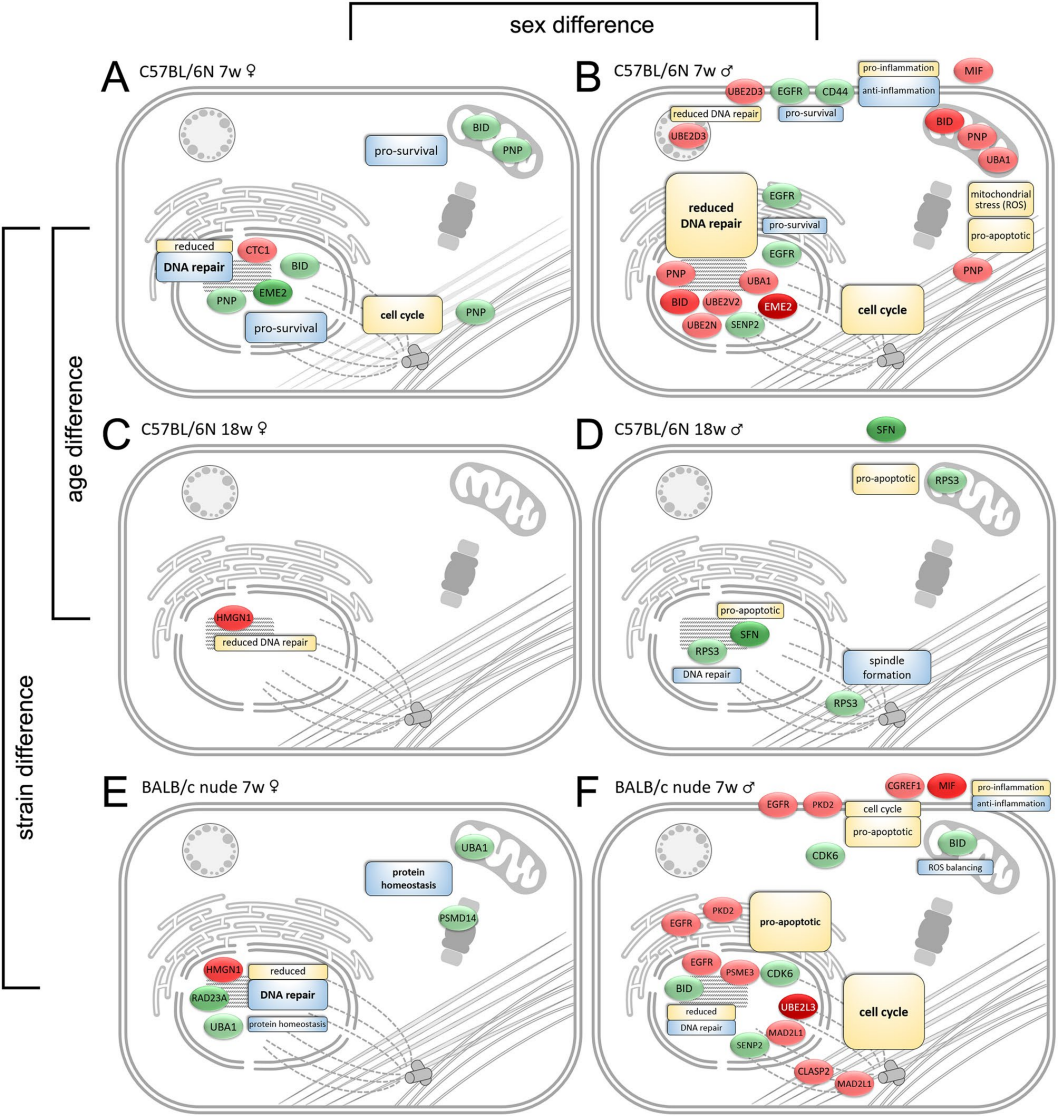
Cellular localization and outcome of differentially abundant proteins associated with DNA damage and repair pathways. Proteins with differential abundance (|FC|≥ 3) in blood plasma 24 h after 0.5 Gy total body irradiation were enriched with Gene Ontology terms for DNA damage and repair processes. Cellular localization according to UniProt (please note different cellular localizations for certain proteins.) Functional outcome inferred from Gene Ontology terms and protein function according to UniProt. (See SI Appendix, Table S8 for further details on functions, interactions, and references.) Panels (**A**–**F**) illustrate protein functions and outcomes for each investigated group (see respective header). Green oval, protein is overabundant; red oval, protein is underabundant; note fold change is visualized by color intensity. Blue rectangles, activation of cellular function/outcome; yellow rectangle, inhibition of cellular function/outcome. 7w, 7-weeks-old (juveniles); 18w, 18-weeks-old (adults); ♀, female; ♂, male. Please note that presented data is normalized to respective unirradiated control groups. Drawings were produced with Microsoft PowerPoint v16.0.4266.1001 and Adobe Photoshop CS5 Portable v12.0.

To determine age and sex effects for DNA damage and repair associated proteins (|FC| cutoff ≥ 3), we performed in-depth pathway-specific analysis using first tier databases and original entries instead of second-tier curated or commercially available pipelines (Fig. 7). Strong qualitative and quantitative differences were found between juvenile and adult mice and between the sexes (Fig. 7A–D). Strain-specific effects, however, were only marginal (cf. Fig. 7E,F). Notably, males showed a larger number of over- or underabundant proteins compared with females irrespective of age or strain. Male juvenile black mice (EGFR, MIF, BID, SENP2, CD44, UBE2N, PNP, UBE2D3, UBE2V2, UBA1, EME2) and male (juvenile) nude mice (BID, CDK6, CGREF1, CLASP2, EGFR, MAD2L1, MIF, PKD2, PSME3, SENP2, UBE2L3) each had 11 differentially abundant proteins with dominating underabundance (Fig. 7B,F); in contrast, female juvenile black mice (BID, CTC1, PNP, EME2) and female (juvenile) nude mice (PSMD14, RAD23A, UBA1, HMGN1) each showed 4 differentially abundant proteins with dominating overabundance (Fig. 7A,E). At adulthood, the number of proteins was distinctly reduced for both black females (HMGN1) and males (SFN, RPS3) (cf. Fig. 7C,D). (For according FC values, please refer to SI Appendix, Fig. S2). Interestingly, sex effects appeared to translate to the spatial dimension of protein abundance as inferred by canonical localization: only male mice, irrespective of age or strain, showed abundance changes in proteins localized in the plasma membrane or extracellular region. Moreover, only juvenile black males showed responses associated with the endosome, whereas only adult black males and (juvenile) nude males showed responses associated with the centrosome.

Consequently, the *inferred cellular outcomes* such as *DNA repair* or *inflammatory responses* differed distinctly between sex and age groups. Comparing juvenile and adult (black) females (cf. Fig. 7A,C), only a minor reduction of DNA repair was shared between both ages, yet conferred by different proteins (CTC1 or HMGN1). In contrast, juvenile females also showed activation of *DNA repair*, *cell cycle arrest*, and *pro-survival processes* while adults showed no other responses. A similar age-dependent trend was found in (black) males (cf. Fig. 7B,D): juveniles showed a general anti-survival outcome with reduced *cell cycle arrest and DNA damage response*, while only two proteins contributed to *pro-survival* and *anti-inflammation* (EGFR, CD44); in contrast, adults showed an activation of *DNA repair* and *cell cycle progression*, with only a minor *pro-apoptotic response* (SFN). Interestingly, strain did not have a pronounced qualitative or quantitative impact on *cellular outcome*–neither in females (cf. Fig. 7A,E) nor males (cf. Fig. 7B,F).

### Pooling did not create analytical bias in this setup

**Figure 8:**
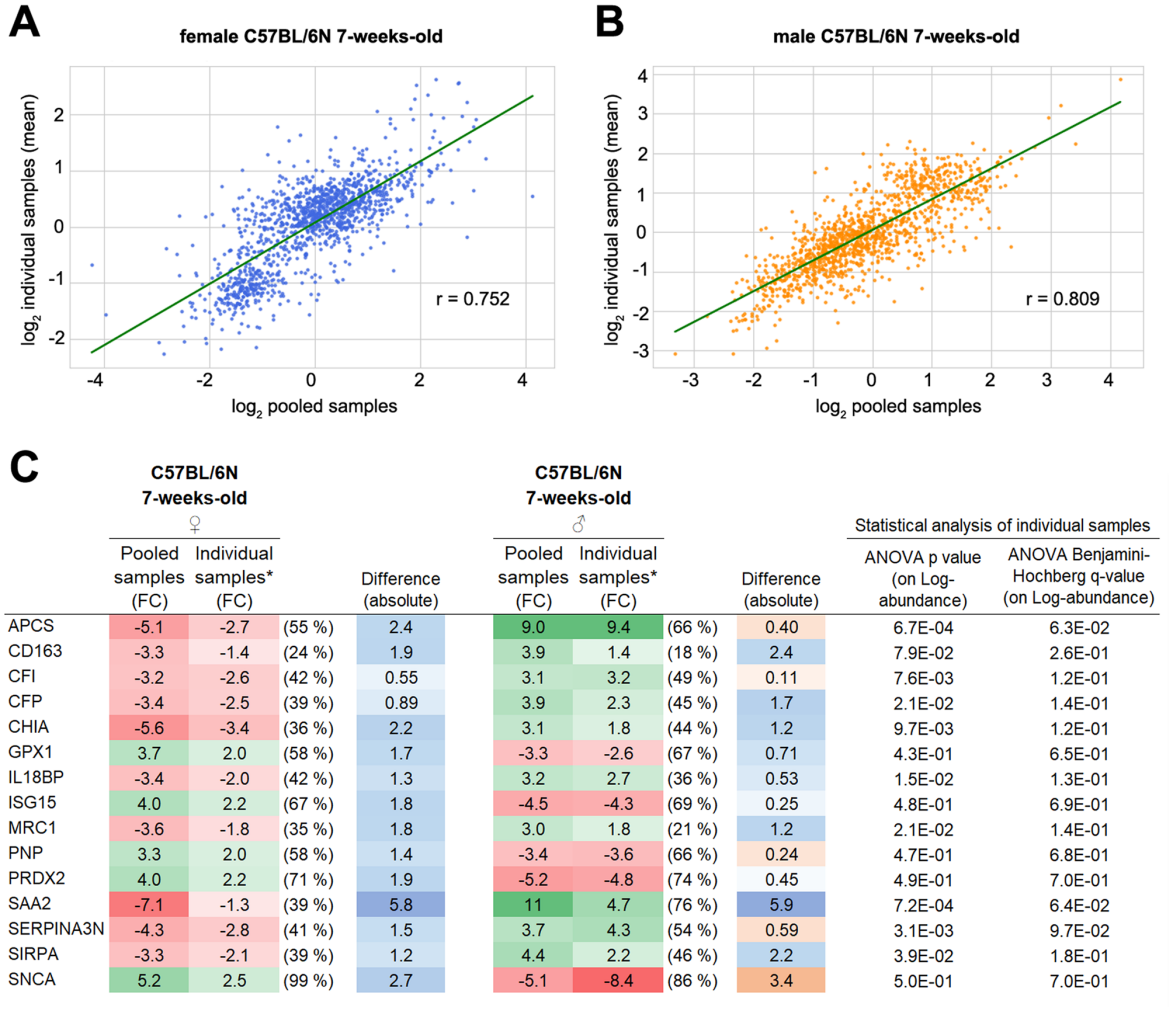
Comparison of pooled and individual sample analysis. The overall correlation between pooled and individual analysis of blood plasma protein 24 h after 0.5 Gy total body irradiation is shown for female juvenile (7-weeks-old) black mice (**A**) and male juvenile (7-weeks-old) black mice (**B**). Note that the data are log-transformed and mean values for individual samples are shown. A representative protein-specific comparison of differential abundance obtained from pooled or individual analysis is shown for 15 randomly selected proteins with immune associated functions (**C**). Note that fold change (FC) is expressed in linear space. *Mean value; standard deviation (SD, parenthesis) for individual sample analysis is given as mean SD value for irradiated and control groups. Blue and orange indicate that relative abundance is higher or lower in pooled analysis than in individual analysis, respectively. Please note that presented data is normalized to respective unirradiated control groups.

We investigated the effect of pooling by global correlation analysis and direct FC comparison for a representative set of proteins (Fig. 8). The correlation between the pooled and individual sample analysis for juvenile black female mice was good with a Pearson’s r = 0.752 (Fig. 8A); for juvenile black male mice, the correlation was even higher with a Pearson’s r = 0.809 (Fig. 8B). For FC comparison, we randomly selected 15 proteins from the pool of immune associated proteins (Fig. 8C). In general, the mean (absolute) FC values from individual analysis were somewhat lower than from pooled analysis while overall trends were reproduced. Few proteins (APCS, CFI, PNP, SERPINA3N, and SNCA) diverged only slightly from this trend. SAA2 was the only protein with a pronounced difference in FC value with 5.5-fold more underabundance obtained from pooled data.

## Discussion

The untargeted proteomic study design allowed us to assess age and sex effects across a wide range of cellular functions and specific IR-associated responses. The high-throughput screening clearly demonstrated that the quality and extent of age and sex effects is endpoint-dependent and should not be generalized. In addition, our pipeline identified analytical bias when using data enrichment. When GO term enrichment was used to identify DNA damage and repair associated proteins, responses were moderate in black male mice but undetected in nude male mice. Manual pathway analysis, on the other hand, yielded similar results for both groups. This finding advises caution and advocates cross-validation of data enrichment with other analytical methods. Total body irradiation was chosen to irradiate all tissues homogeneously and allow for multiple sampling of irradiated tissues in agreement with the 3Rs Reduction principle^42^. The moderate absorbed dose of 0.5 Gy was chosen to exclude potential low-dose effects that may be exclusive to that regimen (typically up to 0.1 Gy^43^), while being low enough to be of relevance for a wide range of risk exposure scenarios. The proteomic response was analyzed 24 h after irradiation to match early monitoring applications. Blood (plasma) was chosen in context with diagnostics and minimally-invasive molecular biomarkers. Blood plasma contains a complex protein mixture mainly synthesized by the liver; but also bone marrow, the spleen, and general tissue cells can contribute to its formation and composition^44^. As such, the observed differential abundance changes may result in large part from ionizing radiation-induced damage to the hematopoietic system, but potentially also reflect responses in other tissues. Nevertheless, interpretation bias may result from overrepresentation of immune-associated proteins upon IR exposure. This may not necessarily mean a strong response in immune processes at this dose level, but rather reflect the specific makeup of plasma protein, which (excluding albumin) consists in large part of globulins and other immune-related proteins.

Pooling was found highly comparable to individual sample analysis, although a minor artefact on dynamic range was observed. Our analytical approach included immunodepletion of seven highly abundant plasma proteins in order to increase the depth of the proteomic analysis. Immunodepletion allows to achieve broad coverage in the LC–MS-based analysis of plasma^45^; however, it is an additional sample preparation step and a potential source of additional analytical bias. The parallel analysis of pooled and individual samples showed the reproducibility of the immunodepletion as a crucial part of the workflow in agreement with literature^46^. We achieved good correlation between the abundance patterns from pooled and individual samples, with the Pearson correlation coefficients reaching 0.75–0.81. Accordingly, we conclude that the extra cost of including both sexes or additional age groups in study designs can be mitigated by pooled analysis in biomarker screening studies.

Regarding the observed strain effects, BALB/c nude mice are immunodeficient since they lack a thymus and are unable to produce T-cells. Immune-related IR responses were thus expected to be impaired and were indeed found to be largely suppressed in nude mice. Interestingly, responses in DNA damage and repair pathways (manual analysis) were comparable between black and nude mice at this dose level, although the BALB/c background is known to be DNA repair deficient. It should also be noted that sex as a variable did not appear to contribute to the strain effects per se, which implied that additional (convoluted) sex bias between strains may be negligible.

In the bulk response, sex effects were marginal compared with the pronounced age impact across the proteome. Although hierarchical clustering indicated a separation by sex, this was mainly due to a single cluster of overabundant proteins shared among all female groups. This cluster consisted of low-abundance change proteins which can be assumed to have less impact on cellular responses. (Accordingly, low-abundance change proteins are filtered in functional analyses and this sex-specific cluster would not carry regulatory relevance.) Combining age and sex into one screening cohort, the analysis yielded only two differentially abundant proteins in all groups. Alpha hemoglobin stabilizing protein (AHSP) showed stable IR response across age, but inverted abundance change (over vs. under) between the sexes. With prior knowledge of expected abundance change, it may qualify as a unisex biomarker candidate. AHSP acts as a chaperone during erythroid cell development^47^. In both *Mus musculus* and *Homo sapiens*, AHSP is expressed in (mainly) bone marrow and blood^47,48^. As such, the biomarker candidate could reflect radiation injury to the hematopoietic system. Serum amyloid A-2 protein (SAA2), a major acute phase reactant^49^, showed large FC range across age and sex to the same stressor and its applicability as a pan-group biomarker was rejected. The very low yield of uniage (or unisex) biomarker candidates strongly supports that screening for age-specific and sex-specific biomarkers is the more feasible approach. Our data suggests that previously identified radiation biomarker candidates may not be translatable across age or sex and we reason that age- and sex-specific validation is paramount.

Overall, the study clearly showed that age-based differences in IR-induced responses outweigh sex-based differences for most analytical endpoints–at least in the investigated age binning.

In theory, a stronger IR response may indicate increased radiosensitivity. Conversely, a stronger (early) IR response might increase e.g. DNA damage recognition and repair and thus reduce risk of malignancies. The increased responses in juveniles supported the former reasoning (juveniles are more radiosensitive than adults according to established knowledge), whereas increased responses in males supported the latter (in general, males are perceived as less radiosensitive than females). Follow-up studies are needed to correlate long-term outcome to this early regulatory response. Regarding sex, the difference in functional regulation between males and females strongly suggests the feasibility of sex-specific immune- or apoptosis-based targets for radioprotectors and mitigators. Concerning age, recent work on age-dependent regulation of mitochondrial apoptosis showed that many tissues (e.g. the heart and kidneys) in post-natal individuals are primed for apoptosis, whereas adult tissues are apoptosis refractory with low expression of the apoptotic machinery^50^. Assuming a similar apoptosis-primed trend for the juvenile mice studied here, reduced DNA repair might be an effect of damaged cells instead undergoing apoptosis due to priming and high expression of the apoptotic machinery. However, studies show that the hematopoietic system (including peripheral blood mononuclear cells) is still primed for apoptosis in adults^50^. Accordingly, age effects from apoptotic priming may be low in blood but could be a significant contributor to IR-responses in other non-hematopoietic tissues.

Proteins for *cell cycle regulation*, *immune responses,* or *DNA damage and repair* that were shared between age, sex, or strain pairs were much less frequent than unique proteins for these processes. Among the shared proteins, only few were identified that may serve as uniage biomarkers of IR-induced responses in females (GMNN and REG3B) and males (SNCA). GMNN (geminin DNA replication inhibitor) is a negative regulator of cell cycle and proliferation; during S-phase, it is involved in suppressing DNA replication and inhibits assembly of the pre-replicative complex until late mitosis^51,52^. A common cellular outcome in cell cycle regulation, however, was not supported by DNA damage and repair pathway analysis. REG3B (regenerating islet-derived protein 3-beta) is a bactericidal C-type lectin involved in the defense responses of the innate immune system^53^. SNCA (alpha-synuclein) is mainly abundant in the presynaptic terminals of brain neurons interacting with phospholipids and proteins^54,55^. Hence, the detection in blood may have come from cranial radiation exposure and SNCA crossing the brain-blood barrier. SNCA may be a potential tissue-specific blood-based biomarker. Nevertheless, since small concentrations are found in other tissues as well, the matter of tissue-specificity needs to be validated. While GMNN, REG3B, and SNCA may be understood as candidates for uniage exposure biomarkers, their dynamic range in dose–response indicated low sensitivity and challenges their applicability. Moreover, the overwhelming dissimilarity of specific protein regulation made a strong case for separate panels of age- and sex-specific radiation biomarkers. It is interesting to note that more proteins with quite similar FC values were identified across strains in females: the multi-ligand integral membrane protein CD36 (cluster of differentiation 36)^56^, the protein-conjugating ISG15 (Interferon-stimulated gene 15)^57^, and the anti-oxidant enzyme PRDX2 (Peroxiredoxin-2)^58^. This finding underlined that strain effects can be negligible for specific endpoints (if only for a given sex).

The qualitative differences in IR-induced responses between age and sex were further highlighted by inferring cellular outcome from DNA damage and repair-associated proteins. As opposed to a mere increase or decrease in response intensity of the same mechanism, juvenile females and males appeared to initiate different signaling cascades. Although both shared suppression of cell cycle progression, females showed activated DNA repair and pro-survival mechanisms whereas males shifted towards pro-apoptosis with mitochondrial stress and reduced DNA repair. In addition, inflammatory regulators, which were not detected in females, appeared to compete over activation and suppression of immune responses. At adult age, sex effects were diminished and, overall, understood as low-to-negligible at this dose level. To the best of our knowledge, these results are the first demonstration of age-dependent sex effects of IR-induced DNA damage and repair. Across strains, the inferred cellular outcome appeared to be rather similar and additional convoluted sex effects were not apparent. Notably, similar outcome was achieved via different mechanisms, as no differential protein levels were shared across strains for either sex. This further strengthened the finding that strain bias in IR-induced responses is a complex matter and should not be generalized across pathways without critical assessment.

## Conclusion

Age and sex had a strong impact on the quality and extent of the proteomic response to moderate IR exposure. Strain-bias may create additional error depending on the investigated endpoint. The very low yield of shared protein regulation implied that unisex or uniage biomarker candidates may not be feasible. Age- and sex-specific screening should be implemented to ensure robustness and diagnostic power of biomarker candidates and to reduce uncertainty and increase the success rate of radiation therapy and intervention. Treatment planning and risk assessment that consider age and sex as essential features are cost-efficient improvements of current practice and a necessary step towards personalized medicine.

## Materials and methods

### Animal procedures and irradiation

Age effects were investigated using 7-weeks-old (‘juvenile’) and 18-weeks-old (‘adult’) C57BL/6N (‘black’) mice (Charles River; Sulzfeld, Germany). Sex effects were investigated using female (♀) and male (♂) mice for both age groups. Sex effects were also investigated in conjunction with strain including juvenile male and female BALB/c nude (‘nude’) mice (CAnN.Cg-Foxn1nu/Crl; Charles River; Sulzfeld, Germany). In total, the study cohort consisted of six irradiated groups (5 mice/group) and six non-irradiated control groups (4–5 mice/group) with matching age, sex, and strain. Group size was chosen based on the accepted minimum number required for proteomics analysis. The study protocol was approved by the Ethical Committee for Animal Research at University of Gothenburg, Gothenburg, Sweden. All animal procedures were carried out in accordance with the ethical approval and reported in in accordance with ARRIVE guidelines.

Animals were allowed to acclimate to the research facility for at least six days upon delivery. Animals were housed in individually ventilated cages and had access to chow and water ad libitum. Mice were total body irradiated with 0.5 Gy absorbed dose from 15 MV nominal photon energy. (For detailed information on the irradiation procedure, please refer to SI Appendix, Supplemental Information Text S1) Control groups were mock-treated following all handling procedures except placing in box for irradiation. Twenty-four hours after irradiation (or mock treatment), the animals were anesthetized (pentobarbitalnatrium vet., i.p.; Apotek Produktion and Laboratorier AB; Sweden) and killed via cardiac puncture. Blood was collected and was stored at − 80 °C until downstream processing. The proteomic analysis was performed using group-wise pooled samples (analysis 1). In order to validate pooling, proteomic analysis was also performed for individual (non-pooled) plasma samples (analysis 2) from irradiated and control groups of female and male juvenile black mice, i.e. for four groups.

### Proteomics analysis

#### Sample preparation

The proteomic analysis was performed using group-wise pooled samples (analysis 1). In order to validate pooling, proteomic analysis was also performed for individual (non-pooled) plasma samples (analysis 2) from irradiated and control groups of female and male juvenile black mice, i.e. for four groups. A comprehensive description of sample preparation procedures is supplied in SI Appendix, Supplemental Information Text S1. In summary, analysis 1 was prepared by mixing equal volumes of plasma from each sample in a group to achieve representative pooled samples. The global pooled reference sample was prepared by mixing equal volumes from all samples. Analysis 2 was prepared by mixing equal volumes from the sample that belonged to the respective groups. Immunodepletion was performed using the Seppro mouse spin column kit (SEP110; Sigma-Aldrich; St Louis, MO, USA) according to the manufacturer’s instruction. Subsequently, the samples were processed according to the modified filter-aided sample preparation (FASP) method^59^. The immunodepleted plasma samples were processed and the resulting peptides were labelled using the TMT 10plex isobaric reagents according to manufacturer’s instructions (Thermo Scientific).

#### Liquid chromatography–mass spectrometry analysis

A comprehensive description of LC–MS analysis is supplied in SI Appendix, Supplemental Information Text S1. Briefly, all samples were analyzed on an Orbitrap Fusion Tribrid mass spectrometer (Thermo Fisher Scientific). For the analysis of the group-wise pooled dataset (analysis 1), the mass spectrometer (MS) was interfaced with an Easy-nLC 1000 liquid chromatography system (Thermo Fisher Scientific). For analysis 2 samples, the MS was interfaced with an Easy-nLC 1200 liquid chromatography system (Thermo Fisher Scientific). Precursor MS scans were performed at 120,000 resolution; MS^3^ spectra were detected in the Orbitrap at 60,000 (analysis 1) or 50,000 (analysis 2) resolution.

### Data analysis

#### Data availability

The mass spectrometry proteomics data have been deposited to the ProteomeXchange Consortium via the PRIDE partner repository with the dataset identifier PXD015859^60^.

#### Proteomic data analysis

A comprehensive description of data analysis is supplied in SI Appendix, Supplemental Information Text S1. Briefly, peptide and protein identification and quantification were performed using Proteome Discoverer version 1.4 (Thermo Fisher Scientific). The files were searched using Mascot 2.3 or 2.5.1 (Matrix Science; London, United Kingdom) against the SwissProt database with taxonomy *Mus musculus* version 2015/04 (16,714 sequences) for analysis 1 and version 2017/11 (16,951 sequence) for analysis 2. The TMT reporter ions were identified in the MS^3^ HCD spectra with a mass tolerance of 3 mmu and minimum intensity threshold of 2000; the resulting reporter abundance values for each sample were normalized on protein median in Proteome Discoverer 1.4. Differential protein abundance between irradiated and respective non-irradiated control groups was expressed as fold change (FC) values by calculating abundance ratios in linear space.

#### Hierarchical cluster analysis

Hierarchical clustering was performed using the heatmap.2 function of the gplots package in the R statistical computing environment version 3.6.0 (https://www.R-project.org/)^61^. Data cohorts were pre-processed by selecting several FC intervals to assess relative distance with regard to low (|FC|< 3), high (3 ≤|FC|< 10), or very-high (|FC|≥ 10) abundance change or a combination thereof as specified.

#### Enrichment and functional analysis

Gene Ontology (GO) term enrichment (http://geneontology.org/) was performed on differentially abundant proteins (|FC|≥ 3). REViGO (http://revigo.irb.hr/) was used to remove redundant GO terms and visualize respective cellular functions in semantic similarity-based treemaps^62^. An additional functional categorization for selected hallmarks of radiation responses based on GO terms, i.e. *immune response*, *oxidative stress*, *apoptotic cell death, other stress responses*, *cell cycle regulation*, *DNA damage and repair*, and *chromatin organization* (see SI Appendix, Tables S2–S7 for complete protein lists and respective categorizations).

A manually curated pathway analysis was performed for differentially abundant proteins (|FC|≥ 3) associated with GO terms for *DNA damage and repair* based on references supplied by UniProt (https://www.uniprot.org/) and functional associations in the GO database; cellular localization was adapted from UniProt. Comprehensive information on functions, interactions, and references are given in SI Appendix, Table S8. Cellular outcome, i.e. induction or inhibition of processes, was inferred from these data in conjunction with protein over- or underabundance in respective pathways.

## Supplementary Information

Supplementary Information.
