# Supplementary material for: Age and sex effects across the blood proteome after ionizing radiation exposure can bias biomarker screening and risk assessment
Source: Sci Rep. 2022 Apr 29;12:7000. doi: 10.1038/s41598-022-10271-3 (PMC9055069; doi:10.1038/s41598-022-10271-3)
Supplement: Supplementary file 1 — Supplementary Information. [file 41598_2022_10271_MOESM1_ESM.pdf]

SI Appendix

The Supplementary Information consists of the following:

Supplementary Information Text (Methods)	p. 2–5
Figure S1	p. 6–8
Figure S2	p. 9
Table S1	p. 10
Table S2	p. 11
Table S3	p. 12
Table S4	p. 13
Table S5	p. 14
Table S6	p. 15
Table S7	p. 16
Table S8	p. 17

Supplementary Information Text

Irradiation procedure

The animals (5 mice/group) were simultaneously total body irradiated with 0.5 Gy absorbed dose to water using a Varian linear accelerator with 15 MV nominal photon energy (Varian Medical Systems, Palo Alto, CA, USA). All irradiations were performed between 5.00 pm and 5.50 pm. The mice were placed in a polystyrene box. The box was covered with 15 mm water-equivalent bolus material. Thus, the mice were positioned at the dose maximum of the beam in the isocenter (1000 mm). A 250×250 mm^2 photon field was employed during the irradiations. The exposure time for dose delivery was 5 seconds. The mice were placed closely together in the polystyrene box to avoid air gaps and to obtain a relatively uniform absorbed dose distribution in each mouse.

Sample Preparation for Proteomic Analysis

For the group-wise pooled quantitation analysis (analysis 1), the representative pooled samples for each group were prepared by mixing equal volumes of plasma from each sample in the respective groups; the global pooled reference sample was prepared by mixing equal volumes from all samples. The reference pooled sample for the non-pooled individual analysis (analysis 2) was prepared by mixing equal volumes from the sample that belonged to the respective groups.

An aliquot of 5.3 µl from each group-wise pooled sample and the global reference sample (analysis 1), or from each individual sample and reference sample (analysis 2), was immunodepleted using the Seppro mouse spin column kit (SEP110; Sigma-Aldrich; St Louis, MO, USA) according to the manufacturer's instruction. Briefly, the plasma samples were loaded onto the spin column in dilution buffer, incubated at room temperature with shaking for 15 min, the depleted sample (eluate 1) was eluted from the spin columns by centrifugation at 2,000 rpm, and the spin columns were washed with 500 µl of dilution buffer (eluate 2), the eluates were combined to yield the depleted sample. Subsequently, the samples were processed according to the modified filter-aided sample preparation (FASP) method (1). In short, the depleted samples were reduced by DL-dithiothreitol (DTT) at the final concentration of 100 mM, reduced samples were diluted 1:4 by volume with 8 M urea, transferred onto the Nanosep 30k Omega filters (Pall Corporation, Ann Arbor, MI) and

washed 2 times with 200 µl of 8 M urea. Alkylation of the reduced cysteine side chains was performed with 10 mM methyl methanethiosulfonate (MMTS) diluted in digestion buffer (1% sodium deoxycholate (SDC), 50 mM TEAB) for 30 min at room temperature and the filters were then repeatedly washed with digestion buffer. Trypsin in digestion buffer was added (500 ng for analysis 1 or 800 ng for analysis 2) and the sample was incubated at 37 °C overnight, then another 500/800 ng portion of trypsin was added followed by the incubation for 2 h (analysis 1) or 3 h (analysis 2). Digested peptides were collected at 10,000 rpm for 20 min, followed by a wash with 20 µl of the digestion buffer and centrifugation at 10,000 rpm for 20 min. In short, the immunodepleted plasma samples were reduced with DL-dithiothreitol, alkylated with methyl methanethiosulfonate, digested with trypsin in presence of 1% sodium deoxycholate. The resulting peptides were labelled using the TMT 10plex isobaric reagents according to the manufacturer's instructions (Thermo Scientific), combined into one TMT set, concentrated using vacuum centrifugation and SDC was removed by acidification with 10% trifluoroacetic acid (TFA) and subsequent centrifugation at 13,000 rpm for 10 min.

The combined TMT-labeled samples were fractionated into 40-42 primary fractions by basic-pH reversed-phase chromatography (bRP-LC) using an ÄKTApurifier (Amersham Pharmacia Biotech AB; Uppsala, Sweden) or a Dionex Ultimate 3000 UPLC system (Thermo Fischer Scientific). All separations were performed using a reversed-phase XBridge BEH C18 column (3.5 µm, 3.0 x 150 mm, Waters Corporation) and a gradient from 0 to 90% acetonitrile in 10 mM ammonium formate (pH 10) at 400 µl/min. The primary fractions were pooled into 20 final fractions, evaporated on speedvac and reconstituted in 3% acetonitrile, 0.2% formic acid for analysis.

Liquid Chromatography-Mass Spectrometry Analysis

All samples were analyzed on an Orbitrap Fusion Tribrid mass spectrometer (Thermo Fisher Scientific). For the analysis of the group-wise pooled dataset (analysis 1), the mass spectrometer (MS) was interfaced with an Easy-nLC 1000 liquid chromatography system (Thermo Fisher Scientific); solvent A was 0.2% formic acid (FA) in water and solvent B was 0.2% FA in acetonitrile. Peptides were trapped on an in-house packed 3 cm pre-column, and separated on an analytical column (75 µm x 30 cm, both backed with Reprosil-Pur C18 material, particle size 3 µm, Dr. Maisch) using the linear gradient from 5% to 25% B in 45 min, from 25% to 80% B in 5 min, 80% B for 10 min, at 300 nL/min flow rate. For analysis 2

samples, the MS was interfaced with an Easy-nLC 1200 liquid chromatography system (Thermo Fisher Scientific); solvent A was 0.2% formic acid (FA) in water and solvent B was 0.2% FA in 80% acetonitrile. Peptides were trapped on an Acclaim Pepmap 100 C18 trap column (100 µm x 2 cm, particle size 5 µm, Thermo Fischer Scientific) or separated on an analytical column (75 µm x 30 cm, particle size 3 µm, Reprosil-Pur C18, Dr. Maisch) using the linear gradient from 5% to 35% B in 75 min, from 35% to 100% B in 5 min, 100% B for 10 min, at 300 nL/min flow rate.

Precursor MS scans were performed at 120,000 resolution; the most abundant precursors with charges 2 to 7 were selected for fragmentation over the 3 s cycle time, fragmented by collision induced dissociation (CID) at 30% (analysis 1) or 35% (analysis 2) collision energy, and the MS^2 spectra were detected in the ion trap followed by the synchronous isolation of the 5 most abundant MS^2 fragment ions and fragmentation by higher-energy collision dissociation (HCD); the resulting MS^3 spectra were detected in the Orbitrap at 60,000 (analysis 1) or 50,000 (analysis 2) resolution.

Data analysis

Data Availability

The detailed proteomic sample preparation protocol and the LC-MS analysis parameters are described in the Electronic Supplementary Information. The mass spectrometry proteomics data have been deposited to the ProteomeXchange Consortium via the PRIDE partner repository with the dataset identifier PXD015859 (2).

Proteomic data analysis

Peptide and protein identification and quantification were performed using Proteome Discoverer version 1.4 (Thermo Fisher Scientific). The files were searched using Mascot 2.3 or 2.5.1 (Matrix Science; London, United Kingdom) against the SwissProt database with taxonomy *Mus musculus* version 2015/04 (16714 sequences) for analysis 1 and version 2017/11 (16951 sequence) for analysis 2. Trypsin with no missed cleavage was used as a cleavage rule, precursor tolerance was set to 5 ppm and MS^2 fragment tolerance was set to 500 mmu (analysis 1) or 600 mmu (analysis 2). Mono-oxidation on methionine was set as a variable modification, cysteine methylthiolation, TMT-6 label on lysine and peptide N-termini were set as fixed modifications. Percolator was used for the peptide-spectrum match (PSM) validation with the strict false discovery rate (FDR) threshold of 1%.

The TMT reporter ions were identified in the MS^3 HCD spectra with a mass tolerance of 3 mmu, and the resulting reporter abundance values for each sample were normalized on protein median in Proteome Discoverer 1.4

Differential protein abundance between irradiated and respective non-irradiated control groups was expressed as fold change (FC) values by calculating abundance ratios in linear space. All subsequent analyses were performed on filtered data.

References

1. Wiśniewski JR, Zougman A, Nagaraj N, Mann M. Universal sample preparation method for proteome analysis. *Nat Methods.* 2009;6:359–362.

2. Perez-Riverol Y, Csordas A, Bai J, Bernal-Llinares M, Hewapathirana S, et al. The PRIDE database and related tools and resources in 2019: improving support for quantification data. *Nucleic Acids Res*. 2019;47(D1):D442–D450.

Panel A. C57BL6N 7-weeks-old ♀

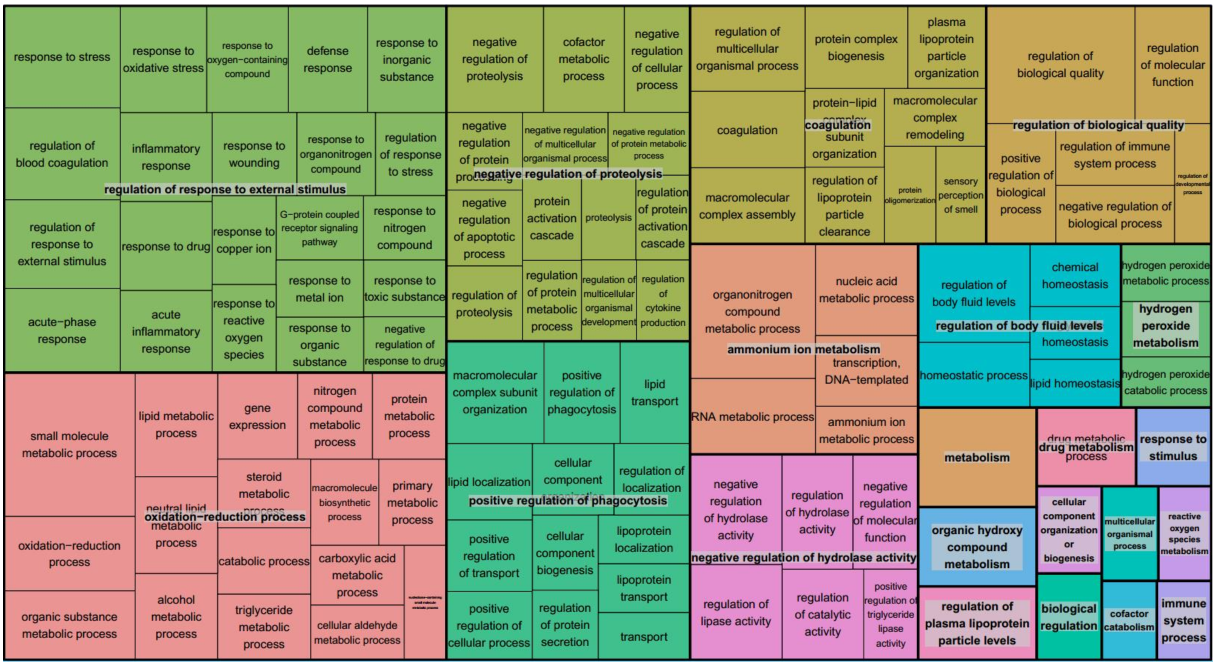

Panel B. C57BL6N 7-weeks-old ♂

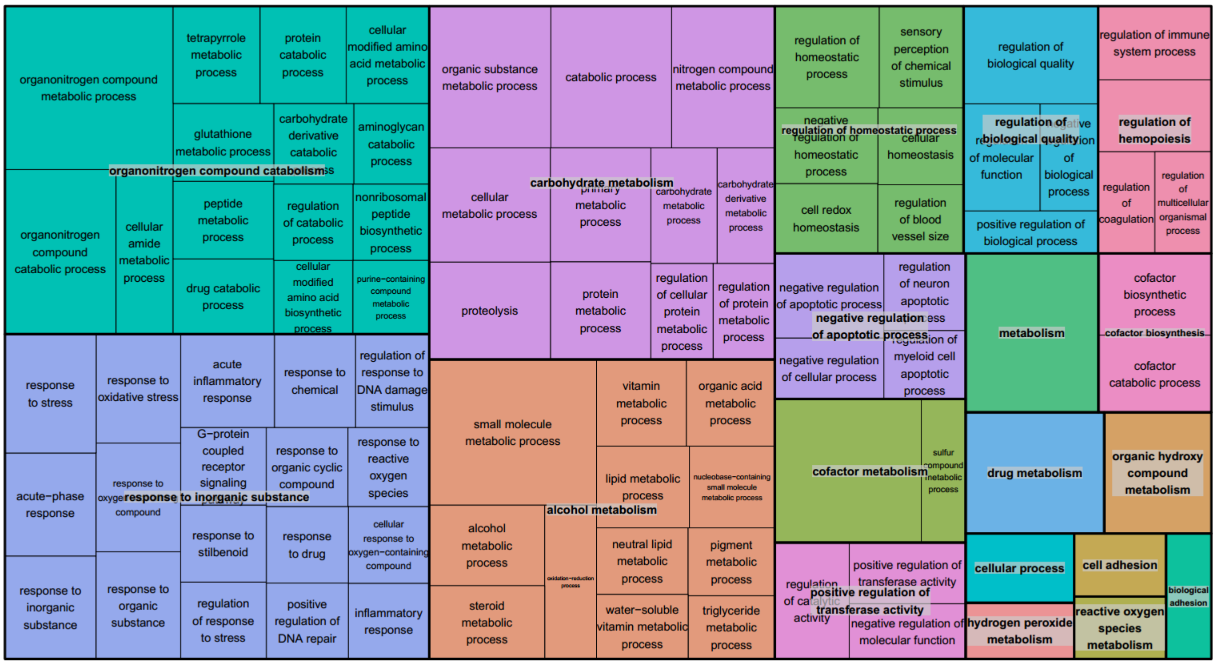

Panel C. C57BL6N 18-weeks-old ♀

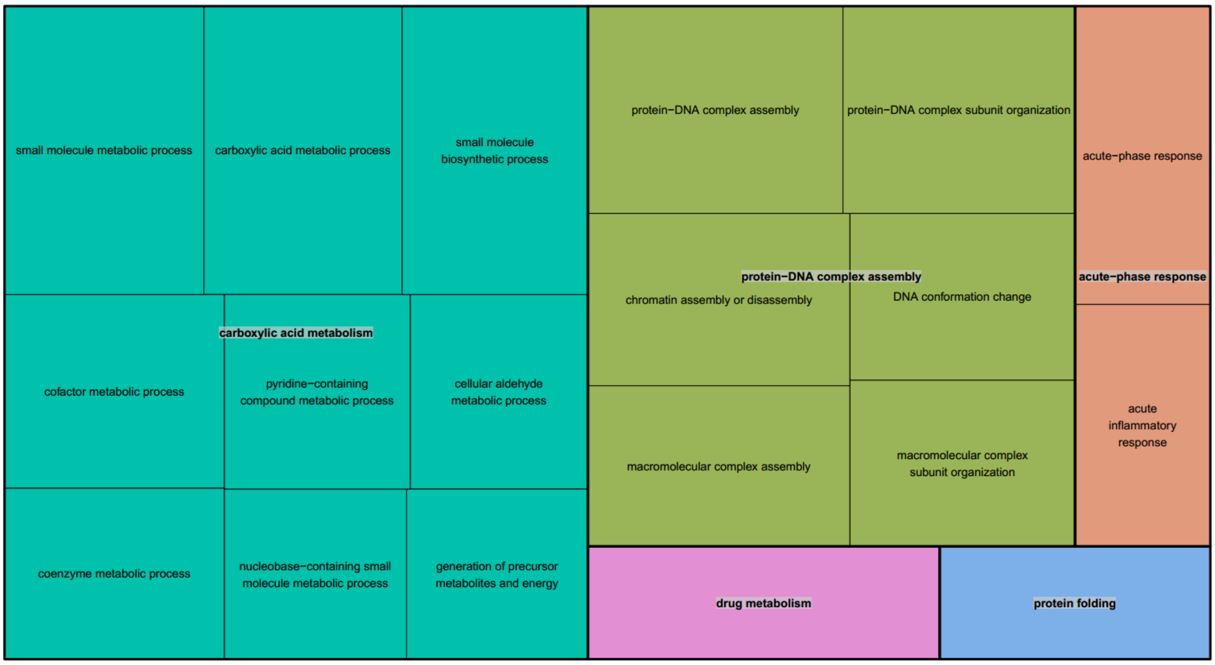

Panel D. C57BL6N 18-weeks-old ♂

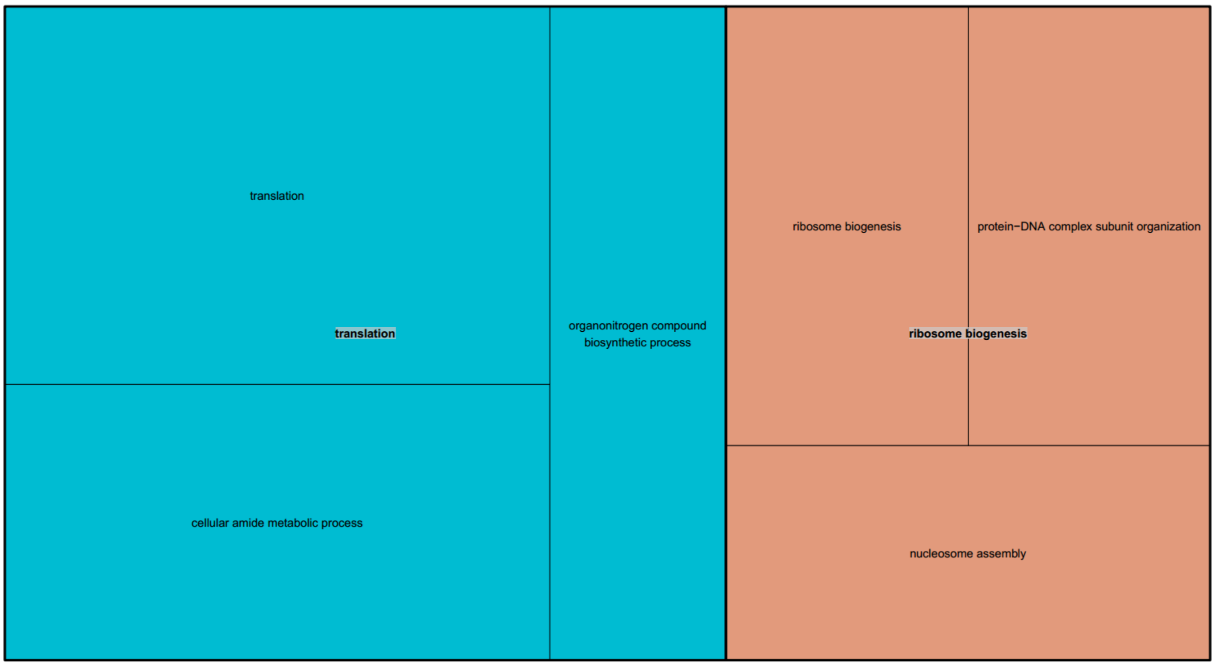

Panel E. BALB/c 7-weeks-old ♀

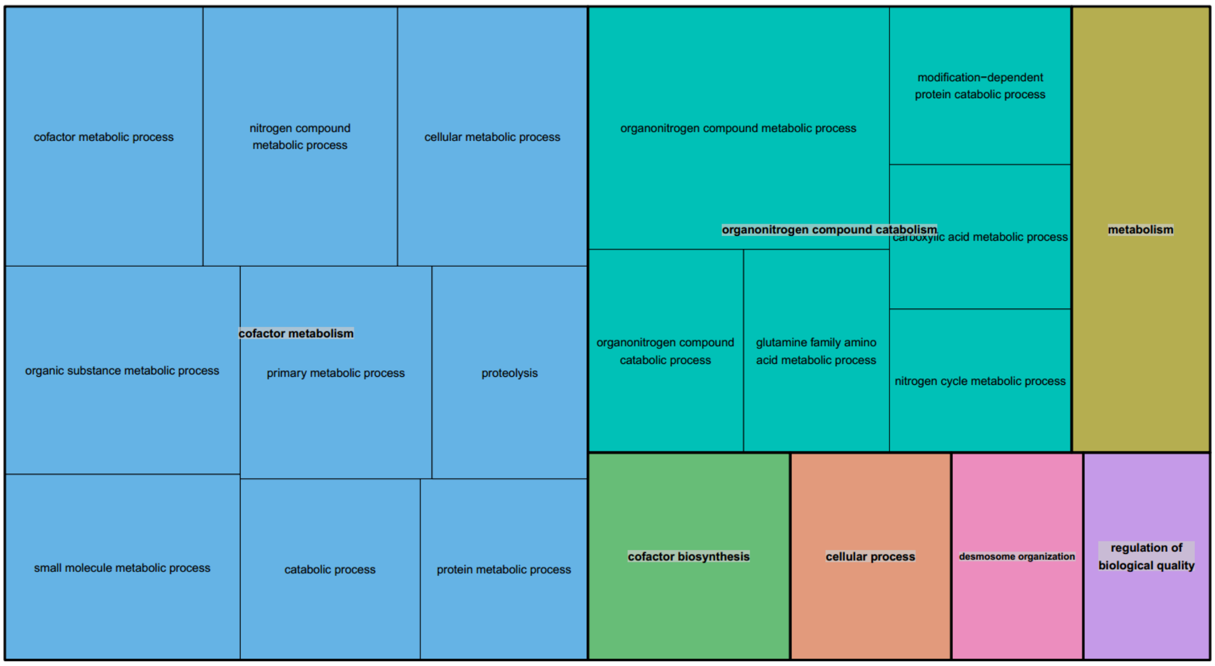

Panel F. BALB/c 7-weeks-old ♂

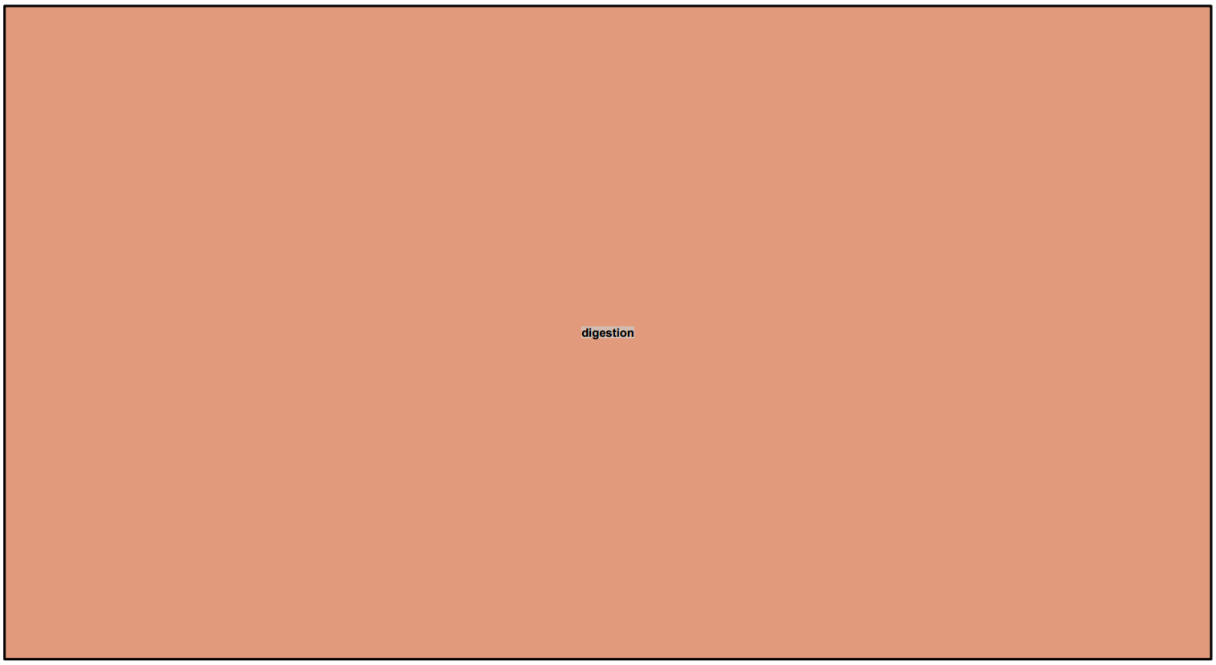

Figure S1. Detailed information on functional associations from enrichment analysis. Gene Ontology (GO) term enrichment analysis (http://geneontology.org/) was performed on differentially abundant proteins with fold change |FC| ≥ 3. REViGO (http://revigo.irb.hr/) was used to remove redundant GO terms and visualize respective cellular functions in the semantic similarity-based treemaps. Please note Panels A-F.

A

C57BL/6N			
7-weeks-old ♀		7-weeks-old ♂	
	FC		FC
BID	3.2	EGFR	3.9
CTC1	-3.9	MIF	-3.8
PNP	3.3	BID	-4.9
EME2	6.6	SENP2	3.5
		CD44	3.6
		UBE2N	-3.2
		PNP	-3.4
		UBE2D3	-3.0
		UBE2V2	-3.4
		UBA1	-3.0
		EME2	-10.0

B

C57BL/6N			
18-weeks-old ♀		18-weeks-old ♂	
	FC		FC
HMGN1	-5.8	SFN	5.0
		RPS3	3.0

C

BALB/c nude			
7-weeks-old ♀		7-weeks-old ♂	
	FC		FC
PSMD14	3.9	BID	3.5
RAD23A	5.1	CDK6	3.9
UBA1	3.2	CGREF1	-3.0
HMGN1	-5.4	CLASP2	-3.4
		EGFR	-3.8
		MAD2L1	-3.4
		MIF	-4.9
		PKD2	-3.2
		PSME3	-3.0
		SENP2	3.6
		UBE2L3	-10.0

Figure S2. Fold change values of differentially abundant proteins associated with DNA damage and repair pathways. The fold change (FC) values of all proteins with |FC| ≥ 3 that are associated with Gene Ontology (GO) terms (http://geneontology.org/) for DNA damage & repair are listed. Comprehensive information on functions, interactions, and references are given in Table S8.

Table S1. Number of proteins associated with radiation-induced responses

Group			Functional category (number of proteins)						
Strain	Age	Sex	Immune response	Oxidative stress	Apoptotic cell death	Other stress responses	Cell cycle regulation	DNA damage & repair	Chromatin organization
C57BL/6N	7-weeks-old	female ♀	45	24	25	5	6	4	4
C57BL/6N	7-weeks-old	male ♂	34	19	30	14	12	11	4
C57BL/6N	18-weeks-old	female ♀	8	7	6	4	2	1	9
C57BL/6N	18-weeks-old	male ♂	6	4	8	0	2	2	8
BALB/c nude	7-weeks-old	female ♀	9	12	12	10	1	4	9
BALB/c nude	7-weeks-old	male ♂	5	0	0	2	0	0	1

Differentially abundant proteins with fold change (FC) values |FC|≥ 3 were subjected to enrichment analysis using Gene Ontology terms (http://geneontology.org/). Based on GO annotations, proteins were grouped into functional categories representing hallmarks of ionizing radiation-induced responses. The number of proteins associated with respective cellular functions is shown for each group.

Table S2. List of proteins associated with hallmark responses induced by ionizing radiation in C57BL/6N 7-weeks-old female mice

				Stress response				Cell cycle regulation	DNA integrity	
**Accession**	**Protein ID**	**Synonym**	**Fold change**	**Immune response**	**Oxidative stress**	**Apoptotic cell death**	**Other**	**Cell cycle regulation**	**DNA damage**	**Chromatin organization**
Q6GQT1	A2M	A2MP	-4.2	immune response						
P61922	ABAT	GABAT	3.4		oxidative stress					
P11859	AGT	SERPINA8	-3.0			apoptotic cell death				
P29699	AHSG	FETUA	-3.1	immune response						
P24549	ALDH1A1	AHD-2, AHD2, ALDH1	4.5			apoptotic cell death				
P47738	ALDH2	AHD-1, AHD1	6.6		oxidative stress	apoptotic cell death				
P12246	APCS	PTX2 SAP	-5.1	immune response						
P09813	APOA2		-4.2	immune response	oxidative stress					
P51910	APOD		-3.5	immune response	oxidative stress					
Q01339	APOH	B2GP1	-5.1			apoptotic cell death				
P70444	BID		3.2			apoptotic cell death		cell cycle regulation	DNA damage	
Q9ES30	C1QTNF3	CORS26, CTRP3	-3.5	immune response						
P06683	C9		-3.3	immune response						
P16015	CA3	CAR3	4.6		oxidative stress					
P24270	CAT	CAS-1, CAS1	9.4		oxidative stress	apoptotic cell death				
Q2VLH6	CD163	M130	-3.3	immune response						
Q08857	CD36		3.6	immune response	oxidative stress	apoptotic cell death				
Q9QWK4	CD5L	AIM, API6	-3.1	immune response		apoptotic cell death				
Q91X79	CELA1 ELA1		5.4	immune response						
Q9CZW2	CENPN		5.9							chromatin organization
Q61129	CFI IF		-3.2	immune response						
P11680	CFP	PFC	-3.4	immune response						
Q8R1U2	CGREF1	CGR11	-18.8					cell cycle regulation		
Q61362	CHI3L1	BRP39, CHIL1	-3.1	immune response		apoptotic cell death				
Q91XA9	CHIA	CHIA1	-5.6	immune response		apoptotic cell death				
P70194	CLEC4F	CLECSF13, KCLR	-3.9	immune response						
Q9JHH6	CPB2	TAFI	-3.3				other			
P14847	CRP	PTX1	-3.1	immune response	oxidative stress					chromatin organization
P07141	CSF1	CSFM	-3.2	immune response						
Q5SUQ9	CTC1		-3.9					cell cycle regulation	DNA damage	
E9Q557	DSP		-3.1				other			
Q56A04	EME2		6.6						DNA damage	
O88513	GMNN		8.3					cell cycle regulation		
P11352	GPX1		3.7	immune response	oxidative stress	apoptotic cell death	other			
O70325	GPX4		3.5		oxidative stress					
P43276	HIST1H1B	H1F5	-3.4							chromatin organization
Q9Z0M9	IL18BP	IGIFBP	-3.4	immune response	oxidative stress					
Q61730	IL1RAP		-3.2	immune response						
Q64339	ISG15	G1P2, UCRP	4.0	immune response						
P26262	KLKB1	KLK3 PK	-3.9	immune response						
P42703	LIFR		-3.3	immune response		apoptotic cell death				
Q5S006	LRRK2		-3.6		oxidative stress	apoptotic cell death				
P24527	LTA4H		6.1							
Q9Z1B5	MAD2L1	MAD2A	3.2			apoptotic cell death		cell cycle regulation		
P39039	MBL1		-4.5	immune response						
Q60805	MERTK	MER	-3.4	immune response		apoptotic cell death				
P21956	MFGE8		-3.1	immune response		apoptotic cell death				
Q61830	MRC1		-3.6	immune response						
P26928	MST1	HGFL	-3.4			apoptotic cell death				
Q64669	NQO1	DIA4, NMO1, NMOR1	3.0		oxidative stress	apoptotic cell death				
Q6P9R2	OXSR1	OSR1	-4.2		oxidative stress					
Q3UR32	P2RX3		4.2		oxidative stress		other			
Q9CQ60	PGLS		3.3		oxidative stress					
Q8VCS0	PGLYRP2	PGLYRPL, PGRPL	-3.2	immune response						
Q60963	PLA2G7	PAFAH	-4.8	immune response	oxidative stress					
P23492	PNP	NP, PNP1	3.3	immune response		apoptotic cell death			DNA damage	
Q91XF0	PNPO		3.6		oxidative stress					
Q61171	PRDX2	TDPX1, TPX	4.0	immune response	oxidative stress	apoptotic cell death				
P33587	PROC		-3.0	immune response		apoptotic cell death				
Q812A5	PRR5	PROTOR1	-3.8					cell cycle regulation		
P35230	REG3B	PAP PAP1	-5.7	immune response						
P47968	RPIA	RPI	3.2		oxidative stress					
P05367	SAA2		-7.1	immune response						
P59110	SENP1	SUPR2	-3.6			apoptotic cell death				
P29621	SERPINA3C	KLKBP	-5.0	immune response						
P07759	SERPINA3K	MCM2, SPI2	-4.8	immune response						
Q03734	SERPINA3M		-4.8	immune response						
Q91WP6	SERPINA3N	SPI2	-4.3	immune response						
P32261	SERPINC1	AT3	-6.2							
Q61247	SERPINF2	PLI	-3.3	immune response						
P97290	SERPING1	C1NH	-5.0	immune response						
P50404	SFTPD	SFTP4	-15.7	immune response						
P97797	SIRPA	BIT, MYD1, PTPNS1, SHPS1, SIRP	-3.3	immune response						
P48962	SLC25A4	ANC1, ANT1	-6.0			apoptotic cell death				
O55042	SNCA SYN		5.2	immune response	oxidative stress	apoptotic cell death				chromatin organization
P08228	SOD1		3.7		oxidative stress	apoptotic cell death	other			
Q62351	TFRC	TRFR	-4.7	immune response	oxidative stress					
Q9JLT4	TXNRD2	TRXR2	3.5		oxidative stress					

Differentially abundant proteins with fold change (FC) values |FC| ≥ 3 were subjected to enrichment analysis using Gene Ontology (GO) terms (http://geneontology.org/).
Based on GO annotations, proteins were grouped into functional categories representing hallmarks of ionizing radiation-induced responses.

Table S3. List of proteins associated with hallmark responses induced by ionizing radiation in C57BL/6N 7-weeks-old male mice

				Stress response				Cell cycle regulation	DNA integrity	
Accession	Protein ID	Synonym	Fold change	Immune response	Oxidative stress	Apoptotic cell death	Other	Cell cycle regulation	DNA damage	Chromatin organization
P24549	ALDH1A1	AHD-2, AHD2, ALDH1	-3.7			apoptotic cell death				
P12246	APCS	PTX2 SAP	9.0	immune response						
Q01339	APOH	B2GP1	3.4			apoptotic cell death				
Q9D9K3	AVEN		-4.9			apoptotic cell death				
P70444	BID		-4.9			apoptotic cell death		cell cycle regulation	DNA damage	
Q9CY64	BLVRA		-3.6		oxidative stress					
Q02105	C1QC	C1QG	3.1	immune response						
P24270	CAT	CAS-1, CAS1	-5.1		oxidative stress	apoptotic cell death				
P48758	CBR1	CBR	-3.1		oxidative stress					
Q2VLH6	CD163	M130	3.9	immune response						
P15379	CD44	LY-24	3.6	immune response		apoptotic cell death	other		DNA damage	
Q64261	CDK6	CDKN6, CRK2	-4.4	immune response				cell cycle regulation		
P31809	CEACAM1	BGP, BGP1, BGPD	3.5	immune response						
Q9CZW2	CENPN		-7.8							chromatin organization
Q61129	CFI IF		3.1	immune response						
P11680	CFP	PFC	3.9	immune response						
Q8R1U2	CGREF1	CGR11	3.5					cell cycle regulation		
Q91XA9	CHIA	CHIA1	3.1	immune response		apoptotic cell death				
Q8BRT1	CLASP2	KIAA0627	3.6				other	cell cycle regulation		
P09581	CSF1R	CSFMR, FMS	3.1	immune response		apoptotic cell death				
P10605	CTSB		3.9	immune response		apoptotic cell death	other			
Q9WV69	DMTN	EPB4.9, EPB49	-3.4				other			
Q01279	EGFR		3.9		oxidative stress	apoptotic cell death		cell cycle regulation	DNA damage	
Q56A04	EME2		-10.0						DNA damage	
P97494	GCLC	GLCLC	-3.3		oxidative stress	apoptotic cell death	other			
O09172	GCLM	GLCLR	-3.3		oxidative stress	apoptotic cell death				
Q9CPU0	GLO1		-4.1			apoptotic cell death				
O88513	GMNN		-7.5					cell cycle regulation		
P11352	GPX1		-3.3	immune response	oxidative stress	apoptotic cell death	other			
P14220	GYPA		-4.0				other			
P22907	HMBS	UROS1	-3.9	immune response	oxidative stress					
Q91X72	HPX HPXN		5.0	immune response						
P48722	HSPA4L	APG1, HSP4L, OSP94	-3.1				other			
Q9JHJ8	ICOSLG	B7H2, B7RP1, ICOSL	3.4	immune response						
O35664	IFNAR2		3.6	immune response						
Q9Z0M9	IL18BP	IGIFBP	3.2	immune response	oxidative stress					
P27931	IL1R2	IL-1R2, IL1RB	3.4	immune response						
Q64339	ISG15	G1P2, UCRP	-4.5	immune response						
P05532	KIT SL		4.8	immune response		apoptotic cell death				
P15947	KLK1	KLK-6, KLK6	3.5				other			
P11438	LAMP1	LAMP-1	3.3	immune response		apoptotic cell death				
P17897	LYZ1	LZP-S	3.3				other			
Q9Z1B5	MAD2L1	MAD2A	-3.5			apoptotic cell death		cell cycle regulation		
P34884	MIF		-3.8			apoptotic cell death		cell cycle regulation	DNA damage	
Q61830	MRC1		3.0	immune response						
Q64669	NQO1	DIA4, NMO1, NMOR1	-3.6		oxidative stress	apoptotic cell death				
Q60590	ORM1	AGP1, ORM-1	6.6	immune response						
P07361	ORM2	AGP-2, ORM-2	18.0	immune response						
Q6P9R2	OXSR1	OSR1	5.0		oxidative stress					
Q9JK95	PERP	KRTCAP1	7.5			apoptotic cell death				
Q9CQ60	PGLS		-3.0		oxidative stress					
Q9ET66	PI16	CRIPI	5.3							
O35245	PKD2	TRPP2	8.4		oxidative stress		other	cell cycle regulation		
P23492	PNP	NP, PNP1	-3.4	immune response		apoptotic cell death			DNA damage	
Q91XF0	PNPO		-3.8		oxidative stress					
P58389	PPP2R4	PTPA	-4.1			apoptotic cell death				
O88531	PPT1	PPT	4.4			apoptotic cell death				
Q61171	PRDX2	TDPX1, TPX	-5.2	immune response	oxidative stress	apoptotic cell death				
Q64695	PROCR	EPCR	3.4	immune response						
P97372	PSME2	PA28B1	-3.1	immune response						
P61290	PSME3		-3.3			apoptotic cell death		cell cycle regulation		
Q3UZA1	RCSD1	CAPZIP	3.3				other			
P05366	SAA1		19.2	immune response						
P05367	SAA2		10.6	immune response						
P59110	SENP1	SUPR2	3.7			apoptotic cell death				
Q91ZX6	SENP2	SMT3IP2, SUPR1	3.5					cell cycle regulation	DNA damage	
Q91WP6	SERPINA3N	SPI2	3.7	immune response						
P97797	SIRPA	BIT, MYD1, PTPNS1, SHPS1, SIRP	4.4	immune response						
O55042	SNCA SYN		-5.1	immune response	oxidative stress	apoptotic cell death				chromatin organization
P08228	SOD1		-3.0		oxidative stress	apoptotic cell death	other			
Q64105	SPR		-3.4		oxidative stress					
Q9JLT4	TXNRD2	TRXR2	-3.5		oxidative stress					
Q02053	UBA1	SBX, UBE1, UBE1AX, UBE1X	-3.0						DNA damage	
P61079	UBE2D3		-3.0			apoptotic cell death			DNA damage	
P68037	UBE2L3	UBCE7	-3.3					cell cycle regulation		
P61089	UBE2N	BLU	-3.2	immune response					DNA damage	chromatin organization
Q9D2M8	UBE2V2	MMS2, UEV2	-3.4			apoptotic cell death			DNA damage	
Q8R5H1	USP15	KIAA0529	-3.2							chromatin organization
Q8CB27	YOD1		-3.2				other			

Differentially abundant proteins with fold change (FC) values |FC| ≥ 3 were subjected to enrichment analysis using Gene Ontology (GO) terms (http://geneontology.org/).
Based on GO annotations, proteins were grouped into functional categories representing hallmarks of ionizing radiation-induced responses.

Table S4. List of proteins associated with hallmark responses induced by ionizing radiation in C57BL/6N 18-weeks-old female mice

				Stress response				Cell cycle regulation	DNA integrity	
**Accession**	**Protein ID**	**Synonym**	**Fold change**	**Immune response**	**Oxidative stress**	**Apoptotic cell death**	**Other**	**Cell cycle regulation**	**DNA damage**	**Chromatin organization**
P24549	ALDH1A1	AHD-2, AHD2, ALDH1	3.6			apoptotic cell death				
P24270	CAT	CAS-1, CAS1	3.7		oxidative stress	apoptotic cell death				
Q9CZW2	CENPN		3.4							chromatin organization
Q8C196	CPS1		3.2		oxidative stress		other			
P15105	GLUL	GLNS	3.6					cell cycle regulation		
O88513	GMNN		3.1					cell cycle regulation		
P06745	GPI	GPI1	3.6			apoptotic cell death				
P14220	GYPA		3.4				other			
P10922	H1F0	H1FV	-3.4							chromatin organization
P01902	H2-K1	H2-K	-23.3	immune response						
P43276	HIST1H1B	H1F5	-4.9							chromatin organization
P10854	HIST1H2BM		-3.8							chromatin organization
P68433	HIST1H3A	H3A, HIST1H3G, H3.1-221, etc.	-3.8							chromatin organization
P62806	HIST1H4A	HIST1H4B, H4-53, HIST1H4C, etc.	-4.8							chromatin organization
P17095	HMGA1	HMGI, HMGIY	-3.2							chromatin organization
P18608	HMGN1	HMG-14, HMG14	-5.8						DNA damage	chromatin organization
P07901	HSP90AA1	HSP86, HSP86-1, HSPCA	4.2	immune response	oxidative stress	apoptotic cell death	other			
P08113	HSP90B1	GRP94, TRA-1, TRA1	4.4		oxidative stress	apoptotic cell death				
O35343	KPNA4	QIP1	3.0		oxidative stress					
P17897	LYZ1	LZP-S	-15.5				other			
Q99KQ4	NAMPT	PBEF1	4.6		oxidative stress					
Q78ZA7	NAP1L4		3.3							chromatin organization
P07361	ORM2	AGP-2, ORM-2	21.5	immune response						
Q6P9R2	OXSR1	OSR1	-3.4		oxidative stress					
Q9DAK9	PHPT1	PHP14	5.5	immune response						
P58389	PPP2R4	PTPA	4.0			apoptotic cell death				
P35230	REG3B	PAP PAP1	-8.7	immune response						
P05366	SAA1		37.3	immune response						
P05367	SAA2		92.7	immune response						
P97430	SLPI		-6.5	immune response						

Differentially abundant proteins with fold change (FC) values |FC| ≥ 3 were subjected to enrichment analysis using Gene Ontology (GO) terms (http://geneontology.org/).
Based on GO annotations, proteins were grouped into functional categories representing hallmarks of ionizing radiation-induced responses.

Table S5. List of proteins associated with hallmark responses induced by ionizing radiation in C57BL/6N 18-weeks-old male mice

				Stress response				Cell cycle regulation	DNA integrity	
**Accession**	**Protein ID**	**Synonym**	**Fold change**	**Immune response**	**Oxidative stress**	**Apoptotic cell death**	**Other**	**Cell cycle regulation**	**DNA damage**	**Chromatin organization**
Q61335	BCAP31	BAP31	3.5			apoptotic cell death				
Q9CQC6	BZW1		4.5							chromatin organization
Q91X79	CELA1 ELA1		-3.2	immune response						
P30275	CKMT1		3.9			apoptotic cell death		cell cycle regulation		
P43276	HIST1H1B	H1F5	3.9							chromatin organization
Q8CGP6	HIST1H2AH		5.3							chromatin organization
P10854	HIST1H2BM		5.5							chromatin organization
P68433	HIST1H3A	H3A, HIST1H3G, H3.1-221, etc.	5.4							chromatin organization
P62806	HIST1H4A	HIST1H4B, H4-53, HIST1H4C, etc.	5.7							chromatin organization
Q8CGP0	HIST3H2BB	HIST3H2BB-PS	6.7							chromatin organization
P11672	LCN2		-4.3	immune response	oxidative stress	apoptotic cell death				
P48678	LMNA LMN1		3.1		oxidative stress	apoptotic cell death				
Q9Z0Y2	PLA2G1B	PLA2	-4.7	immune response						
P62830	RPL23		3.3							
P27659	RPL3		3.0	immune response						
P62908	RPS3		3.0		oxidative stress	apoptotic cell death			DNA damage	
P05367	SAA2		-4.7	immune response						
O70456	SFN	MKRN3	5.0			apoptotic cell death		cell cycle regulation	DNA damage	
P51881	SLC25A5	ANT2	5.7			apoptotic cell death				
O55042	SNCA SYN		-3.1	immune response	oxidative stress	apoptotic cell death				chromatin organization

Differentially abundant proteins with fold change (FC) values |FC| ≥ 3 were subjected to enrichment analysis using Gene Ontology (GO) terms (http://geneontology.org/).
Based on GO annotations, proteins were grouped into functional categories representing hallmarks of ionizing radiation-induced responses.

Table S6. List of proteins associated with hallmark responses induced by ionizing radiation in BALB/c nude 7-weeks-old female mice

				Stress response				Cell cycle regulation	DNA integrity	
**Accession**	**Protein ID**	**Synonym**	**Fold change**	**Immune response**	**Oxidative stress**	**Apoptotic cell death**	**Other**	**Cell cycle regulation**	**DNA damage**	**Chromatin organization**
Q8BGQ7	AARS		3.2			apoptotic cell death	other			
Q62151	AGER	RAGE	-7.1	immune response	oxidative stress	apoptotic cell death	other			
P24270	CAT	CAS-1, CAS1	5.7		oxidative stress	apoptotic cell death				
Q08857	CD36		4.9	immune response	oxidative stress	apoptotic cell death				
Q61735	CD47		4.6	immune response						
Q9CZW2	CENPN		6.1							chromatin organization
Q8C196	CPS1		4.8		oxidative stress		other			
O89114	DNAJB5	HSC40	-5.0				other			
E9Q557	DSP		-3.4				other			
P54731	FAF1		3.9			apoptotic cell death				
P35550	FBL		-4.2							chromatin organization
P97494	GCLC	GLCLC	4.8		oxidative stress	apoptotic cell death	other			
O09172	GCLM	GLCLR	5.6		oxidative stress	apoptotic cell death				
P14220	GYPA		5.7				other			
P10922	H1F0	H1FV	-4.9							chromatin organization
P15864	HIST1H1C	H1F2	-4.0							chromatin organization
P22907	HMBS	UROS1	4.5	immune response	oxidative stress					
P17095	HMGA1	HMGI, HMGIY	-4.0							chromatin organization
P18608	HMGN1	HMG-14, HMG14	-5.4						DNA damage	chromatin organization
P09602	HMGN2	HMG-17, HMG17	-3.4							chromatin organization
P07901	HSP90AA1	HSP86, HSP86-1, HSPCA	5.1	immune response	oxidative stress	apoptotic cell death	other			
P08113	HSP90B1	GRP94, TRA-1, TRA1	3.9		oxidative stress	apoptotic cell death				
P48722	HSPA4L	APG1, HSP4L, OSP94	3.0				other			
Q64339	ISG15	G1P2, UCRP	3.4	immune response						
P17897	LYZ1	LZP-S	-3.8				other			
P28656	NAP1L1	NRP	4.4							chromatin organization
Q78ZA7	NAP1L4		5.3							chromatin organization
P58389	PPP2R4	PTPA	4.9			apoptotic cell death				
Q61171	PRDX2	TDPX1, TPX	4.2	immune response	oxidative stress	apoptotic cell death				
O35593	PSMD14	PAD1	3.9						Damage and repair	
P54726	RAD23A	MHR23A	5.1						DNA damage	
P47968	RPIA	RPI	3.2		oxidative stress					
P14869	RPLP0	ARBP	3.3	immune response						
Q80UG5	SEPT9	KIAA0991, SINT1	-5.5					cell cycle regulation		
Q9QZI9	SERINC3	AIGP1, DIFF33, TDE1, TMS1	4.0	immune response		apoptotic cell death				
Q9JIM1	SLC29A1	ENT1	7.5		oxidative stress					
Q02053	UBA1	SBX, UBE1, UBE1AX, UBE1X	3.2						DNA damage	

Differentially abundant proteins with fold change (FC) values |FC| ≥ 3 were subjected to enrichment analysis using Gene Ontology (GO) terms (http://geneontology.org/).
Based on GO annotations, proteins were grouped into functional categories representing hallmarks of ionizing radiation-induced responses.

Table S7. List of proteins associated with hallmark responses induced by ionizing radiation in BALB/c nude 7-weeks-old male mice

				Stress response				Cell cycle regulation	DNA integrity	
**Accession**	**Protein ID**	**Synonym**	**Fold change**	**Immune response**	**Oxidative stress**	**Apoptotic cell death**	**Other**	**Cell cycle regulation**	**DNA damage**	**Chromatin organization**
Q91X79	CELA1 ELA1		3.0	immune response						
Q08093	CNN2		3.4	immune response			other			
P08121	COL3A1		-3.3	immune response			other			
O35601	FYB		5.6	immune response						
P39039	MBL1		-3.2	immune response						
P28656	NAP1L1	NRP	3.3							chromatin organization

Differentially abundant proteins with fold change (FC) values |FC| ≥ 3 were subjected to enrichment analysis using Gene Ontology (GO) terms (http://geneontology.org/).
Based on GO annotations, proteins were grouped into functional categories representing hallmarks of ionizing radiation-induced responses.

Table S8. Detailed information on differentially abundant proteins associated with DNA damage and repair pathways

Protein	Protein name	Compartment	Category	Functions	Key interaction with: direct	Key interaction with: indirect (downstream effect on)	References
BID	BH3-interacting domain death agonist	nucleus, mitochondria	DNA damage & repair, cell cycle	balancing mitochondrial ROS; critical pro-survival role in cell cycle arrest	ATM	pro-survival	Gross et al. Cell Death Differ. 2016;23(1):182
		nucleus	DNA damage & repair	ATM effector in the DNA damage response	ATM		Kamer et al. Cell. 2005;122(4):593-603
CD44	CD44 antigen	plasma membrane	immune system (among others)	role in activation, recirculation and homing of T-lymphocytes, hematopoiesis, inflammation, cell migration, and cell-cell adhesion	various proteins	various	Wu et al. Immunity. 2014;41(2):270-82; Naujokas et al. Cell. 1993;74(2):257-68.
CDK6	cyclin-dependent kinase 6	nucleus, cytosol	cell cycle	plays a role in the accumulation of p53 and p130 keeping cells from entering cell division if there is DNA damage, activating pro- apoptotic pathways		p53, p130	Nagasawa et al. Oncogene. 2001;20(23):2889-99.
CGREF1	cell growth regulator with EF hand domain protein 1	extracellular region	cell cycle	can inhibit growth			by similarity
			calcium signaling	mediates cell-cell adhesion in a calcium-dependent manner	calcium signaling		by similarity
CLASP2	CLIP-associating protein 2	cytosol	cell cycle	stabilizing function at the kinetochore which is essential for the bipolar alignment of chromosomes on the mitotic spindle; similar function for microtubules	kinetochore, microtubules		Pereira AL et al. Mol Biol Cell. 2006;17(10):4526-42.
CTC1	CST complex subunit CTC1	nucleus	cell cycle, DNA damage & repair	component of the CST complex proposed to act as a specialized replication factor promoting DNA replication under conditions of replication stress or natural replication barriers such as the telomere duplex.	DNA	DNA replication/damage recovery	Miyake et al. Mol Cell. 2009;36(2):193-206.
				DNA replication/damage recovery: CST complex facilitates recovery from many forms of exogenous DNA damage; involved in several aspects of telomere replication	DNA		Wu et al. Cell. 2012;150(1):39-52.
EGFR	epidermal growth factor receptor	nucleus, ER, plasma membrane	cell cycle	receptor tyroC16:F18sine kinase activates several signaling cascades resulting in positive regulation of cell proliferation and inhibition of apoptosis	adapter proteins (various)	RAS-RAF-MEK-ERK, PI3 kinase-AKT, PLCgamma-PKC and STATs modules; speculated: NF-kappa-B	Fazioli et al. EMBO J. 1993 Oct;12(10):3799-808; Confalonieri et al. J Cell Biol. 2000;150(4):905-12; Peng et al. Nucleic Acids Res. 2010;38(21):7446-57.
EME2	probable crossover junction endonuclease EME2	nucleus	DNA damage & repair, DNA recombination	endonuclease activity: interacts with MUS81 to form a DNA structure-specific endonuclease which cleaves substrates such as 3'-flap structures	MUS81, DNA		by similarity
				DNA double-strand break repair, intra-S DNA damage checkpoint, replication fork processing	DNA		GO annotations
HMGN1	non-histone chromosomal protein HMG-14	nucleus	DNA damage & repair, transcription	binds to nucleosomal DNA altering DNA-histone interaction; may be involved in maintaining transcribable genes in a unique chromatin conformation	DNA		Lim et al. Mol Cell. 2004;15(4):573-84.
				role in transcription-coupled nucleotide-excision repair, chromatin organization, transcription, and UV-B/C responses	DNA		GO annotations
MAD2L1	MAD2 mitotic arrest deficient-like 1	nucleus, cytoskeleton	cell cycle	component of the spindle-assembly checkpoint and required for the execution of the mitotic checkpoint	checkpoint control		by similarity
MIF	macrophage migration inhibitory factor	extracellular region	immune system	pro-inflammatory cytokine involved in the innate immune response and counteracts the anti-inflammatory activity of glucocorticoids			by similarity
						NF-κB	Kim et al. Cell Signal.;34:110-120.
PKD2	polycystin-2	plasma membrane, ER	calcium signaling	component of a homotetrameric or heteromeric (with PKD1) calcium-permeable ion channel activated through Wnt signaling	calcium signaling		Kim et al. Nat Cell Biol. 2016;18(7):752-764.
PNP	purine nucleoside phosphorylase	nucleus, mitochondria, cytoskeleton	DNA damage & repair, immune system, apoptosis, purine metabolism	involved in purine nucleoside salvage; also functional association with DNA damage & repair, immune system, and negative regulation of T cell apoptotic process	ribonucleosides	anti-apoptotic	Uniprot; GO annotations; Jenuth and Snyder. Nucleic Acids Res. 1991;19(7):1708.
PSMD14	26S proteasome non-ATPase regulatory subunit 14	cytosol	DNA damage & repair, cell cycle, apoptosis, proteasome	part of the 26S proteasome maintaining protein homeostasis; thus involved in various processes such as cell cycle progression, apoptosis, and DNA damage repair	proteasome		by similarity
PSME3	proteasome actiA29:C29	nucleus	DNA damage & repair, cell cycle, apoptosis	subunit of the 11S REG-gamma proteasome regulator with anti-apoptotic function and potential role in cell cycle regulation: facilitates the MDM2-p53/TP53 interaction and thus degradation of p53/TP53, which limits its accumulation resulting in inhibition of apoptosis following DNA damage	p53/TP53	anti-apoptotic	by similarity
RAD23A	UV excision repair protein RAD23 homolog A	nucleus	DNA damage & repair	involved in nucleotide excision repair as XPC:RAD23A dimer (NER activity); speculated to be equivalent for Rad23b in global genome nucleotide excision repair (GG-NER)	XPC		Ng et al. Genes Dev. 2003;17(13):1630-45; Okuda et al. DNA Repair (Amst). 2004;3(10):1285-95.
			proteasome	involved in modulation of proteasomal degradation (26S proteasome)	proteasome		by similarity
RPS3	40S ribosomal protein S3	nucleus, mitochondria, cytoskeleton	DNA damage & repair	role in DNA damage repair (also high affinity for 8-oxoG lesions)	DNA, UNG1, APEX1		Kim et al. J Biol Chem. 1995;270(23):13620-9; by similarity
			cell cycle	involved in spindle formation and chromosome movement by regulating microtubule polymerization during mitosis	microtubule regulation		by similarity
			apoptosis	binds to and TP53/p53 and protects from MDM2-mediated ubiquitination; pro-apoptotic function through its role in activating CASP8 (among others)	p53/TP53	pro-apoptotic	Jang et al. FEBS Lett. 2004;560(1-3):81-5.; GO annotations
			transcription	part of the NF-kappa-B p65-p50 complex (binds to the RELA/p65 subunit)	NF-κB		by similarity
SENP2	sentrin-specific protease 2	nucleus	posttranslational modification (sumoylation)	protease in the SUMO pathway	various proteins	Wnt pathway (speculated)	by similarity
			DNA damage & repair	negative regulation of DNA damage response (signal transduction by p53 class mediator)		suppresses DNA damage response	GO annotations
			cell cycle	regulation of G1/S transition of mitotic cell cycle		checkpoint control	GO annotations
SFN	14-3-3 protein sigma	nucleus, extracellular region	signaling, cell cycle	adapter protein implicated in the regulation of various general and specialized signaling pathways	various proteins	general and specialized signaling	by similarity; GO annotations
			apoptosis	intrinsic apoptotic signaling pathway in response to DNA damage		pro-apoptotic	GO annotations
UBA1	ubiquitin-like modifier-activating enzyme 1	nucleus, mitochondria	ubiquitin-proteasome system	plays a key role in the ubiquitin-proteasome system catalyzing the first step in ubiquitin conjugation	proteasome		
			DNA damage & repair	essential for the formation of radiation-induced foci and DNA repair, as well as for responses to replication stress; promotes the recruitment of TP53BP1 and BRCA1 at DNA damage sites		TP53BP1, BRCA1	by similarity
UBE2D3	ubiquitin-conjugating enzyme E2 D3	plasma membrane, endosome	ubiquitin-proteasome system	part of the ubiquitination system	proteasome	NFKBIA proteasomal degradation	by similarity; GO annotations
			DNA damage & repair	involved in the DNA damage tolerance (DDT) pathway by regulating mono- and poly-ubiquitination of PCNA		ubiquitination of PCNA	by similarity; GO annotations
				involved in ubiquitination of DNA lesions by interacting with the BRCA1/BARD1 E3 ligase complex	BRCA1/BARD1 E3 ligase complex	ubiquitination of DNA lesions	by similarity; GO annotations
			apoptosis	plays a role in ubiquitination of p53/TP53 together with the MDM2 and TOPORS E3 ligases	MDM2, TOPORS E3 ligases	ubiquitination of p53/TP53	by similarity; GO annotations
UBE2L3	ubiquitin-conjugating enzyme E2 L3	nucleus	ubiquitin-proteasome system	ubiquitin-conjugating enzyme E2 that specifically acts with HECT-type and RBR family E3 ubiquitin-protein ligases (e.g. PRKN and ARIH1)	E3 ubiquitin-protein ligases (HECT-type and RBR family)	selective degradation of short-lived and abnormal proteins	by similarity; GO annotations
			cell cycle	involved in regulating progression through the cell cycle (down-regulated during the S-phase)		cell cycle progression	by similarity; GO annotations
			transcription	regulates transcriptional activity of nuclear hormone receptors		transcriptional activity (nuclear hormone receptors)	by similarity; GO annotations
UBE2N	ubiquitin-conjugating enzyme E2 N	nucleus	DNA damage & repair	plays a role in error-free DNA repair and contributes to the survival of cells after DNA damage; acts together with the E3 ligases (HLTF and SHPRH) in the 'Lys-63'-linked poly-ubiquitination of PCNA upon genotoxic stress	HLTF, SHPRH	pro-survival	by similarity; GO annotations
			cell cycle	plays a role in regulating cell cycle progression		cell cycle progression	by similarity
			immune system, signaling	interacts with TRIM5 for polyubiquitination which activates the MAP3K7/TAK1 complex resulting in expression of NF-kappa-B and MAPK-responsive inflammatory genes	TRIM5	induces inflammatory genes (via NF-κB, MAPK)	by similarity; GO annotations
			non-canonical ubiquitination	catalyzes the synthesis of non-canonical 'Lys-63'-linked polyubiquitin chains, which dos not lead to protein degradation by the proteasome	various proteins		Cardamone et al. Mol Cell. 2012;46(1):91-104; Lentucci. J Biol Chem. 2017;292(7):2754-2772.
UBE2V2	ubiquitin-conjugating enzyme E2 variant 2	nucleus	DNA damage & repair	plays a role in error-free DNA repair		pro-survival	Franko et al. Biochim Biophys Acta. 2001;1519(1-2):70-7; GO annotations
			non-canonical ubiquitination	has no ubiquitin ligase activity on its own; forms UBE2V2/UBE2N heterodimer to catalyze 'Lys-63'-linked poly-ubiquitination chains, which dos not lead to protein degradation by the proteasome	UBE2N, various proteins		GO annotations
			cell cycle	plays a role in regulating cell cycle progression			GO annotations

Differentially abundant proteins with fold change (FC) values |FC| ≥ 3 were subjected to enrichment analysis using Gene Ontology (GO) terms (http://geneontology.org/).
Based on GO annotations, all proteins that were associated with DNA damage & repair and related cellular functions were used in the pathway analysis.
Accordingly, the table features only protein functions that are relevant for DNA damage recognition & repair and related cellular functions.
Additional functions in normal cell physiology may thus be omitted for clarity.
Protein functions were adapted from Uniprot (www.uniprot.org) and GO annotations (http://geneontology.org); reference *by similarity was* taken from Uniprot (www.uniprot.org).
